# Radiation causes tissue damage by dysregulating inflammasome–gasdermin D signaling in both host and transplanted cells

**DOI:** 10.1371/journal.pbio.3000807

**Published:** 2020-08-06

**Authors:** Jianqiu Xiao, Chun Wang, Juo-Chin Yao, Yael Alippe, Tong Yang, Dustin Kress, Kai Sun, Kourtney L. Kostecki, Joseph B. Monahan, Deborah J. Veis, Yousef Abu-Amer, Daniel C. Link, Gabriel Mbalaviele

**Affiliations:** 1 Division of Bone and Mineral Diseases, Washington University School of Medicine, St. Louis, Missouri, United Sates of America; 2 Division of Oncology, Washington University School of Medicine, St. Louis, Missouri, United Sates of America; 3 Department of Spine Surgery, Honghui Hospital, Xi'an Jiaotong University, Xi'an, Shaanxi, China; 4 Aclaris Therapeutics, Inc., St. Louis, Missouri, United Sates of America; 5 Department of Orthopedic Surgery, Washington University School of Medicine, St. Louis, Missouri, United Sates of America; 6 Shriners Hospital for Children, St. Louis, Missouri, United Sates of America; National Institute of Biological Sciences, CHINA

## Abstract

Radiotherapy is a commonly used conditioning regimen for bone marrow transplantation (BMT). Cytotoxicity limits the use of this life-saving therapy, but the underlying mechanisms remain poorly defined. Here, we use the syngeneic mouse BMT model to test the hypothesis that lethal radiation damages tissues, thereby unleashing signals that indiscriminately activate the inflammasome pathways in host and transplanted cells. We find that a clinically relevant high dose of radiation causes severe damage to bones and the spleen through mechanisms involving the NLRP3 and AIM2 inflammasomes but not the NLRC4 inflammasome. Downstream, we demonstrate that gasdermin D (GSDMD), the common effector of the inflammasomes, is also activated by radiation. Remarkably, protection against the injury induced by deadly ionizing radiation occurs only when NLRP3, AIM2, or GSDMD is lost simultaneously in both the donor and host cell compartments. Thus, this study reveals a continuum of the actions of lethal radiation relayed by the inflammasome-GSDMD axis, initially affecting recipient cells and ultimately harming transplanted cells as they grow in the severely injured and toxic environment. This study also suggests that therapeutic targeting of inflammasome-GSDMD signaling has the potential to prevent the collateral effects of intense radiation regimens.

## Abstract 2

Radiotherapy is a commonly used conditioning regimen for bone marrow transplantation; cytotoxicity limits the use of this life-saving therapy, but the underlying mechanisms remain poorly defined. This study reveals a continuum of the effects of radiation, relayed by the inflammasome–gasdermin D axis, initially affecting host cells and, ultimately, harming transplanted cells.

## Introduction

Total body irradiation (TBI) is used in combination with other therapies as a conditioning regimen for the transplantation of bone marrow, cord blood, or hematopoietic stem cells (HSCs) for patients with hematological malignancies such as acute and chronic leukemia, lymphoma, and myelodysplastic syndrome [1–3]. TBI followed by HSC transplantation is also recommended for the treatment of nonmalignant blood disorders, including hemoglobinopathies, aplastic anemia, and immune deficiencies [3,4]. Thus, TBI-containing conditioning regimens are widely used in the clinic, yet they cause life-threatening injuries and side effects in multiple organs, including the intestine, lungs, kidney, brain, and spleen [2,4–7].

The skeleton is also adversely affected by radiotherapy, as this procedure damages bone extracellular matrix, dysregulates the differentiation and activity of bone-forming cells (osteoblasts) and hematopoietic bone-resorbing cells (osteoclasts [OCs]), and destroys bone marrow hematopoiesis niches, events that, ultimately, cause growth retardation, osteoporosis, and higher fracture risk [8–12]. Thus, the increased survival rates of patients subjected to radiotherapy raise concerns for long-term skeletal complications, which can worsen other morbidities. For example, it was reported that 20% to 50% of geriatric patients (≥65 years) with a hip fracture die within 1 year of the injury [13–17]. Therefore, understanding the mechanisms through which high doses of radiation induce bone toxicity may open avenues for tailored adjuvant therapies to improve the quality of life of the survivors.

TBI is routinely used in preclinical settings as a myeloablative treatment for allogeneic or syngeneic adoptive cell transfer (ACT). High doses of TBI cause massive cell death, thereby generating cues that activate inflammatory pathways, including the inflammasomes, in innate and adaptive immune cells in the allogeneic or syngeneic models. Consistent with this view, the inflammasomes, including those assembled by AIM2 and NLRP3, are implicated in radiation-associated tissue injury [18–22]. Thus, although the inflammasomes are a normal participant in immune responses and tissue repair, their hyperactivation is unhealthy. Yet the extent to which the activities of the inflammasomes in transplanted and/or host cells impact tissue outcomes following ACT in animals conditioned by TBI remains poorly understood.

The inflammasomes are intracellular protein complexes that process pro-interleukin-1β (pro-IL-1β) and pro-IL-18 into IL-1β and IL-18, respectively [23,24]. The inflammasomes also cleave gasdermin D (GSDMD) into N-terminal GSDMD (GSDMD^Nt^) and C-terminal GSDMD (GSDMD^Ct^) fragments; the GSDMD^Nt^ moieties form pores at the plasma membrane through which IL-1β and IL-18 are secreted into the extracellular milieu [25–28]. Although these cytokines are efficiently released by live cells, sustained generation of GSDMD^Nt^ causes cytolysis as the result of excessive pore formation, which compromises the integrity of the plasma membrane [29–31]. This form of cell demise, called pyroptosis, is proinflammatory as lysed cells uncontrollably release not only IL-1β and IL-18 but also danger-associated molecular patterns (DAMPs) such as ATP, high mobility group box 1 (HMGB1), and S100A9 proteins [32–34]; the release of these DAMPs results in the recruitment of immune cells and the perpetuation of inflammation.

In this study, we determined the impact of inflammasome sufficiency or insufficiency in donor and/or host cells on TBI-induced tissue injury in the syngeneic bone marrow transplantation (BMT) mouse model. We find that this procedure causes systemic inflammation and damage to several tissues, including bones and the spleen. These anomalies are significantly reduced when transplanted and recipient cells simultaneously lack NLRP3, AIM2, or GSDMD, but not NLRC4. These findings establish a novel concept whereby the inflammasome pathways mediate collateral effects of high dose of radiation not only in resident cells but also in transferred cells as the result of the toxic environment that this treatment creates in the host.

## Results

### Radiation causes bone injury

The high prevalence of bone loss following radiotherapy [8–12] provided a strong rationale for assessing changes in this tissue in mice exposed to a high dose of radiation. To optimize the TBI/BMT protocol, we leveraged TRAP-tdTomato (tdT) reporter mice in which the *Acp5* promoter drove tdT expression in OC precursors [35], which derived from HSCs [36]. Wild-type (WT; *tdT*^*-*^) mice were subjected to 9 Gy, a clinically relevant dose of radiation [1,11,12]. Because this radiation was lethal, irradiated animals were inoculated with bone marrow cells from *tdT*^*+*^ mice to generate *tdT*^*+*^→*tdT*^*-*^ mice. Conversely, *tdT*^*-*^ cells were injected into irradiated *tdT*^*+*^ mice to obtain *tdT*^*-*^→*tdT*^*+*^ mice. Nonirradiated *tdT*^*+*^ mice and *tdT*^*-*^ mice of the same sex and age served as positive and negative controls, respectively. All nontransplanted, irradiated mice died within 10 days, whereas >95% of transplanted mice overcome the lethality. Flow cytometry analysis revealed that approximately 6% and 2.5% of bone marrow cells were *tdT*^*+*^ cells in nonirradiated *tdT*^*+*^ mice and *tdT*^*+*^→*tdT*^*-*^ mice, respectively, 3 weeks post TBI/BMT (S1A and S1B Fig). These values were higher than the 0.6% background fluorescence signal of *tdT*^*-*^ mice and *tdT*^*-*^→*tdT*^*+*^ mice and consistent with the reported frequency of OC precursors in bone marrow [37–39]. Attempts to confirm the depletion of *tdT*^*+*^ cells by tissue imaging were unsuccessful, likely because of the modest activity of the *Acp5*-*tdT* reporter. In any case, micro–computed tomography (μCT) analysis revealed that all WT→WT mice developed time-dependent loss of bone mass (bone volume/total volume [BV/TV]), associated with increased number and surface of OCs, pronounced adipogenesis, and decreased dynamic indices of bone formation (S1C–S1H Fig). Thus, lethal radiation efficiently depletes host OC precursors, which are replaced by donor counterparts. Although radiation adversely affects bone formation and resorption, this work focuses on the latter phase of bone turnover.

### Radiation damages bones through the NLRP3 and AIM2 inflammasomes, and GSDMD, but not the NLRC4 inflammasome

**Fig 1:**
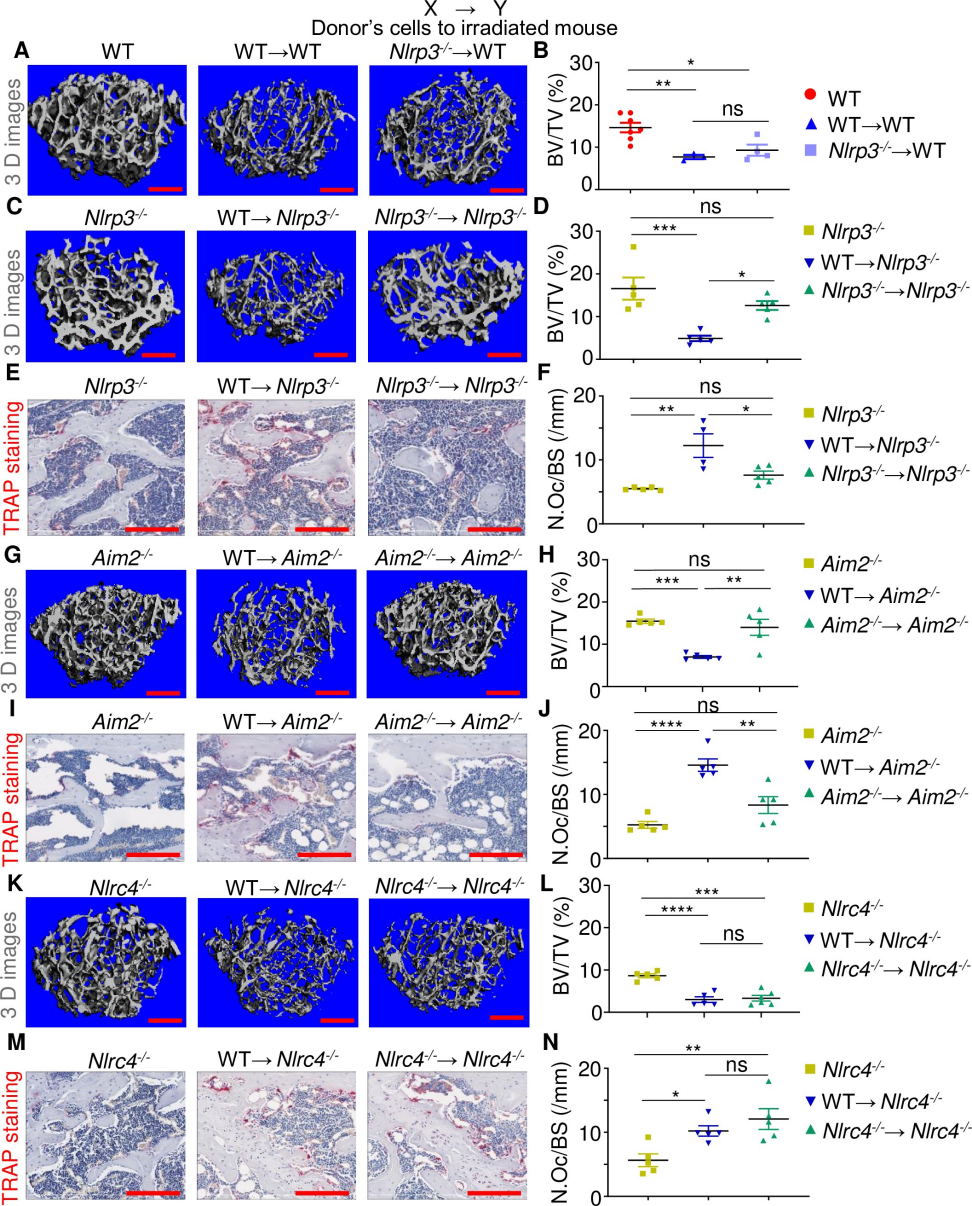
Radiation damages bones through the NLRP3 and AIM2 inflammasomes but not the NLRC4 inflammasome. Three-month-old WT, *Nlrp3*^*-/-*^, *Aim2*^*-/-*^, and *Nlrc4*^*-/-*^ male mice were left untreated or subjected to 9-Gy TBI. Irradiated mice were transplanted with 10^7^ bone marrow cells from 3-month-old WT or null male mice to generate WT→WT, *Nlrp3*^*-/-*^→WT, WT→*Nlrp3*^*-/-*^, *Nlrp3*^*-/-*^→*Nlrp3*^*-/-*^, WT→*Aim2*^*-/-*^, *Aim2*^*-/-*^→*Aim2*^*-/-*^, WT→*Nlrc4*^*-/-*^, and *Nlrc4*^*-/-*^→*Nlrc4*^*-/-*^ mice. The femurs were harvested 3 weeks later and analyzed by μCT. (A, C, G, K) Cross sections of 3D reconstructions. (B, D, H, L) BV/TV. The femurs were also stained for TRAP activity. (E, I, M) TRAP^+^ cells (OCs), stained in red. (F, J, N) N.Oc/BS. The numerical values underlying Fig 1B, 1D, 1F, 1H, 1J, 1L, 1N can be found in S1 Data. Data are mean ± SEM. *P < 0.05, **P < 0.005, ***P < 0.0005, ****P < 0.0001. Scale bar: 200 μm. μCT, micro–computed tomography; BV/TV, bone volume/total volume; N.Oc/BS, OC number/bone surface; ns, not significant; OC, osteoclast; TBI, total body irradiation; WT, wild-type.

Endogenous molecules such as ATP and uric acid released by dying cells are sensed as DAMPs by surrounding live innate cells, leading to the activation of the NLRP3 inflammasome [22,32,34,40]. The massive cell death that radiation caused to host tissues provided a strong rationale for investigating its impact on the NLRP3 inflammasome. WT→WT and *Nlrp3*^*-/-*^→WT male mice exhibited similar bone loss compared to WT controls (Fig 1A and 1B; S2A and S2B Fig). By contrast, relative to WT mice, bone loss, OC number, and surface were significantly attenuated in *Nlrp3*^*-/-*^→*Nlrp3*^*-/-*^ mice compared to WT→*Nlrp3*^*-/-*^ male mice (Fig 1C–1F; S2C and S2D Fig). Thus, loss of NLRP3 simultaneously in donor and recipient cells is necessary to significantly reduce radiation-induced bone loss in host mice.

Self-DNA [18–21] and nucleotide-derived metabolites [41] activate the AIM2 and NLRC4 inflammasomes, respectively, though the NLRP3 inflammasome is also activated by mitochondrial DNA [42–44]. Given the DNA-destabilizing actions of radiation, we determined the extent to which the AIM2 and NLRC4 inflammasomes contributed to radiation-mediated bone injury. Based on the similarity of the phenotype of *Nlrp3*^*-/-*^→WT mice and WT→*Nlrp3*^*-/-*^ mice, *Aim2*^*-/-*^ or *Nlrc4*^*-/-*^ but not WT mice were used as hosts. Although radiation caused bone loss and increased OC formation in WT→*Aim2*^*-/-*^ mice compared to nonirradiated *Aim2*^*-/-*^ mice, these responses were significantly attenuated in *Aim2*^*-/-*^→*Aim2*^*-/-*^ mice (Fig 1G–1J; S2E and S2F Fig). By contrast, osteolysis occurred in WT→*Nlrc4*^*-/-*^ mice comparably to *Nlrc4*^*-/-*^→*Nlrc4*^*-/-*^ mice (Fig 1K–1N; S2G and S2H Fig). Collectively, these results show that TBI causes bone demise through the NLRP3 and AIM2 inflammasomes but not the NLRC4 inflammasome.

**Fig 2:**
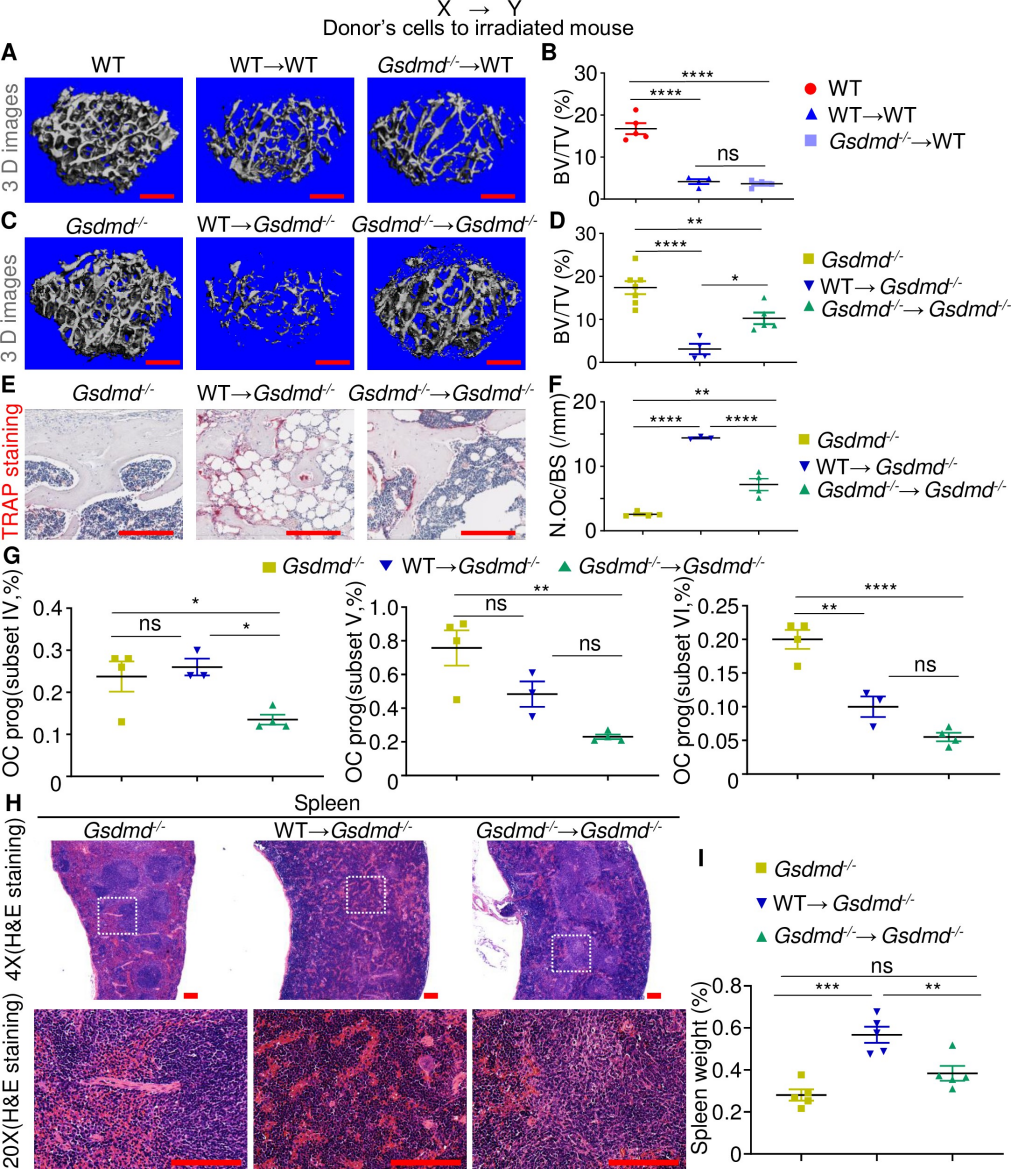
Radiation damages bones and the spleen through GSDMD. Three-month-old WT and *Gsdmd*^*-/-*^ male mice were left untreated or subjected to 9-Gy TBI. Irradiated mice were transplanted with 10^7^ bone marrow cells from 3-month-old WT or *Gsdmd*^*-/-*^ male mice to generate WT→WT, *Gsdmd*^*-/-*^→WT, WT→*Gsdmd*^*-/-*^, and *Gsdmd*^*-/-*^→*Gsdmd*^*-/-*^ mice. Samples were harvested 3 weeks later. The femurs were analyzed by μCT. (A, C) Cross sections of 3D reconstructions. (B, D) BV/TV. The femurs were also stained for TRAP activity. (E) TRAP^+^ cells (OCs), stained in red. (F) N.Oc/BS. Scale bar: 200 μm. The percentage of OC progenitors (“prog”) in bone marrow cells was analyzed by flow cytometry (G; the gating is shown in S4 Fig). (H) H&E staining of the spleen. Scale bar: 100 μm. (I) Percentage of spleen weights. The numerical values underlying Fig 2B, 2D, 2F, 2G, 2I can be found in S1 Data. Data are mean ± SEM. *P < 0.05, **P < 0.005, ***P < 0.0005, ****P < 0.0001. μCT, micro–computed tomography; BV/TV, bone volume/total volume; GSDMD, gasdermin D; H&E, hematoxylin and eosin; N.Oc/BS, OC number/bone surface; ns, not significant; OC, osteoclast; TBI, total body irradiation; WT, wild-type.

We also assessed the degree to which the massive bone loss that occurred in the TBI/BMT model was dependent on GSDMD, a downstream target of the inflammasomes and mediator of pyroptosis [25–28,45]. WT→WT and *Gsdmd*^*-/-*^→WT male mice (Fig 2A and 2B) and female mice (S3A and S3B Fig) lost bone comparably relative to WT controls. Interestingly, bone mass was higher whereas OC number and surface were lower in *Gsdmd*^*-/-*^→*Gsdmd*^*-/-*^ female mice (S3C and S3D Fig) and male mice (Fig 2C–2F; S3E Fig) compared to corresponding WT→*Gsdmd*^*-/-*^ mice. Thus, radiation-associated osteolysis is another pathology that implicates GSDMD besides autoinflammatory and autoimmune disorders [45–47].

**Fig 3:**
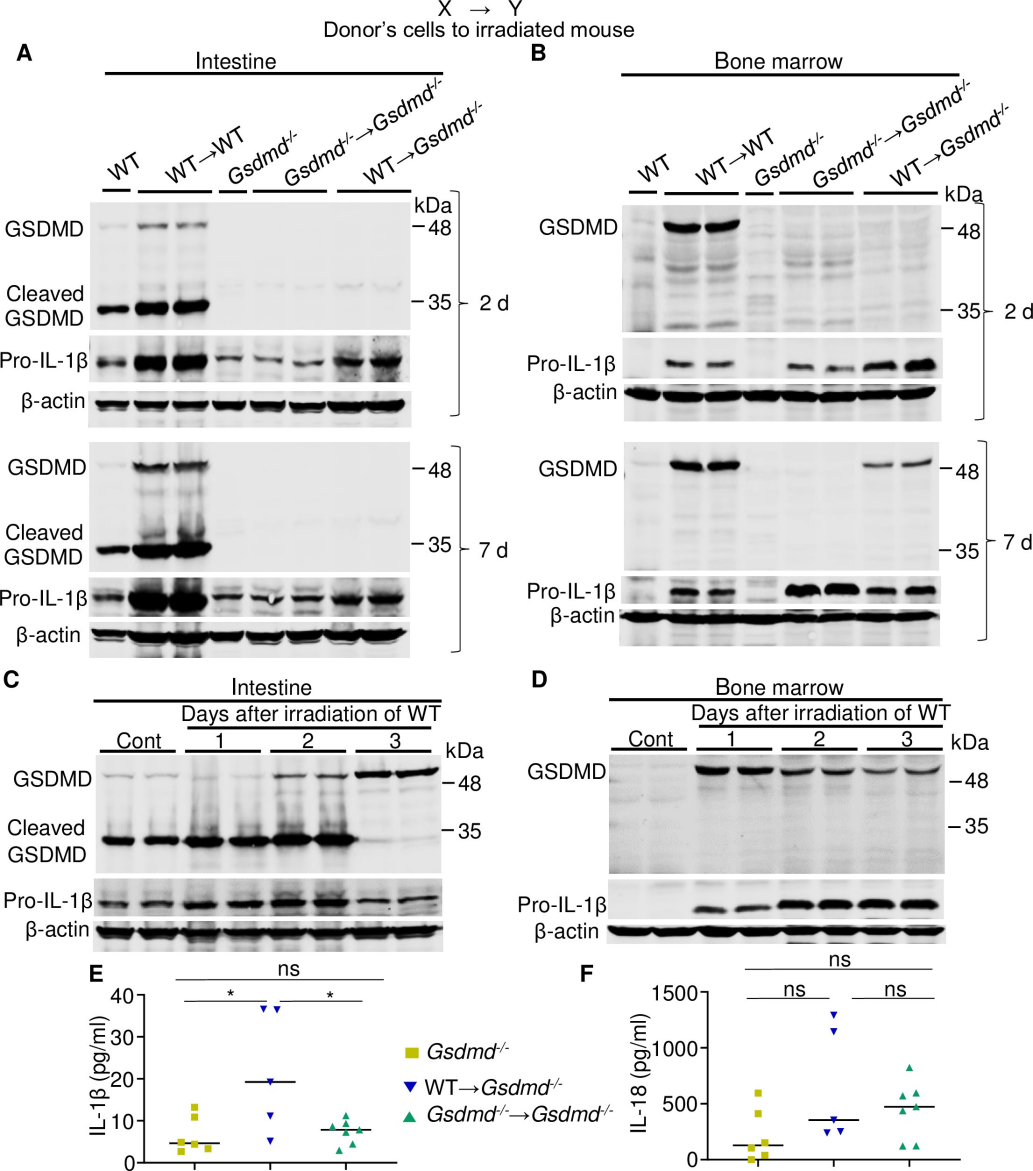
Radiation-induced systemic inflammation correlates with the extent of GSDMD activation. (A, B, E, F) Three-month-old WT or *Gsdmd*^*-/-*^ male mice were left untreated or subjected to 9-Gy TBI. Irradiated mice were transplanted with 10^7^ bone marrow cells from 3-month-old WT or *Gsdmd*^*-/-*^ male mice to generate WT→WT, *Gsdmd*^*-/-*^→*Gsdmd*^*-/-*^, and WT→*Gsdmd*^*-/*^ mice. (C, D) Three-month-old WT male mice were left untreated or subjected to 9-Gy TBI (irradiated mice were not transplanted with bone marrow cells). Samples from 1–2 mice/group were collected 2 or 7 days after TBI/BMT (A, B) or 1, 2, and 3 days post TBI (C, D) and analyzed alongside control (”Cont”) lysates by immunoblotting. (E, F) IL-1β and IL-18 levels in the serum from chimeric mice (3 weeks post TBI/BMT) and control mice. The data underlying this figure may be found in S1 Data and S2 Data. Data are mean ± SEM. *P < 0.05. BMT, bone marrow transplantation; GSDMD, gasdermin D; IL, interleukin; ns, not significant; TBI, total body irradiation; WT, wild-type.

Given the similarity of the effects of NLRP3, AIM2, or GSDMD deficiency, we focused on this pore-forming protein to gain mechanistic insights into the actions of radiation. Flow cytometry analysis revealed that the percentage of the highly osteoclastogenic B220^-^CD3^-^CD11b^-/low^CD115^+^CD117^high^ cells (subset IV) [39,48] was significantly reduced in *Gsdmd*^*-/-*^→*Gsdmd*^*-/-*^ mice compared to WT→*Gsdmd*^*-/-*^ mice (Fig 2G; S4 Fig). A similar trend was observed for B220^-^CD3^-^CD11b^-/low^CD115^+^CD117^int^ cells (subset V) and B220^-^CD3^-^CD11b^-/low^CD115^+^CD117^low^ cells (subset VI), which were also endowed with the OC formation potential [39,48] (Fig 2G). Thus, GSDMD plays a crucial role in the expansion of OC precursors and exuberant bone resorption caused by radiation.

### Radiation-induced tissue injury correlates with the extent of GSDMD activation

Histological assessments of the spleen of *Gsdmd*^*-/-*^ mice showed intact red and white pulp (Fig 2H), similar to WT controls. Remarkably, WT→*Gsdmd*^*-/-*^ mice showed effacement of the normal splenic architecture due to extensive extramedullary hematopoiesis (EMH) that likely was the cause of the increased spleen weights (Fig 2I). By contrast, *Gsdmd*^*-/-*^→*Gsdmd*^*-/-*^ mice displayed significant retention of splenic architecture, with less EMH, corresponding to the smaller spleen size. Consistent with bone outcomes, spleen enlargement was significantly reduced in *Nlrp3*^*-/-*^→*Nlrp3*^*-/-*^ and *Aim2*^*-/-*^→*Aim2*^*-/-*^ but not *Nlrc4*^*-/-*^→*Nlrc4*^*-/-*^ mice compared to WT→*Nlrp3*^*-/-*^, WT→*Aim2*^*-/-*^, and WT→*Nlrc4*^*-/-*^ mice, respectively (S5A–S5C Fig). Thus, inactivation of GSDMD in chimeric mice attenuates the severity of spleen injury caused by high dose of radiation.

We also analyzed the expression and maturation of GSDMD in tissues harvested 2 and 7 days after TBI/BMT. Although the levels of GSDMD were consistently higher in the intestine, bone marrow, spleen, and liver of WT→WT mice compared to WT mice (Fig 3A and 3B; S6A and S6B Fig), cleaved GSDMD was detected only in the intestine. Furthermore, GSDMD was readily detectable in bone marrow and spleen but not in the intestine and liver from WT→*Gsdmd*^*-/-*^ mice 7 days after TBI/BMT (Fig 3A and 3B; S6A and S6B Fig). We observed similar patterns of GSDMD expression and maturation in tissues from nontransplanted irradiated mice (TBI without BMT), harvested on day 1, 2, or 3 post radiation when BMT was dispensable for the survival of mice (Fig 3C and 3D; S6C and S6D Fig). Noticeably, cleaved GSDMD was undetectable in the intestine 3 days after irradiation (Fig 3C), likely as the result of pyroptosis of certain cell populations caused by excessive generation of GSDMD^Nt^ fragment. Collectively, these findings ruled out the scenario that the increase in GSDMD levels in WT→WT tissues relative to WT controls was simply the result of a higher number of cells in chimeric mice. Thus, radiation induces not only the expression of GSDMD but also its activation, responses that affect cell fates in tissue in a context-dependent manner.

Pro-IL-1β levels in the intestine, bone marrow, spleen, and liver were higher in most samples from WT→WT or TBI without BMT compared to nonirradiated WT (Figs 3A–3D; S6A and S6B). Accordingly, the levels of IL-1β measured at the end of the studies (3 weeks post-TBI/BMT) were higher in the serum from WT→*Gsdmd*^*-/-*^ mice compared to *Gsdmd*^*-/-*^→*Gsdmd*^*-/-*^ animals (Fig 3E); a similar trend was noted for serum IL-18 levels (Fig 3F). By contrast, the levels of monocytes, polymorphonuclear cells (PMNs), and lymphocytes in blood (S6E–S6H Fig) and bone marrow (S6I and S6J Fig) at 3 weeks post TBI/BMT were not different between WT→*Gsdmd*^*-/-*^ mice and *Gsdmd*^*-/-*^→*Gsdmd*^*-/-*^ mice. Thus, the absence of GDSMD in chimeric mice results in decreased radiation-induced secretion of IL-1β and IL-18, though the number of inflammatory cells in the blood is unaffected at the time of sampling.

### GSDMD exerts OC lineage autonomous actions

**Fig 4:**
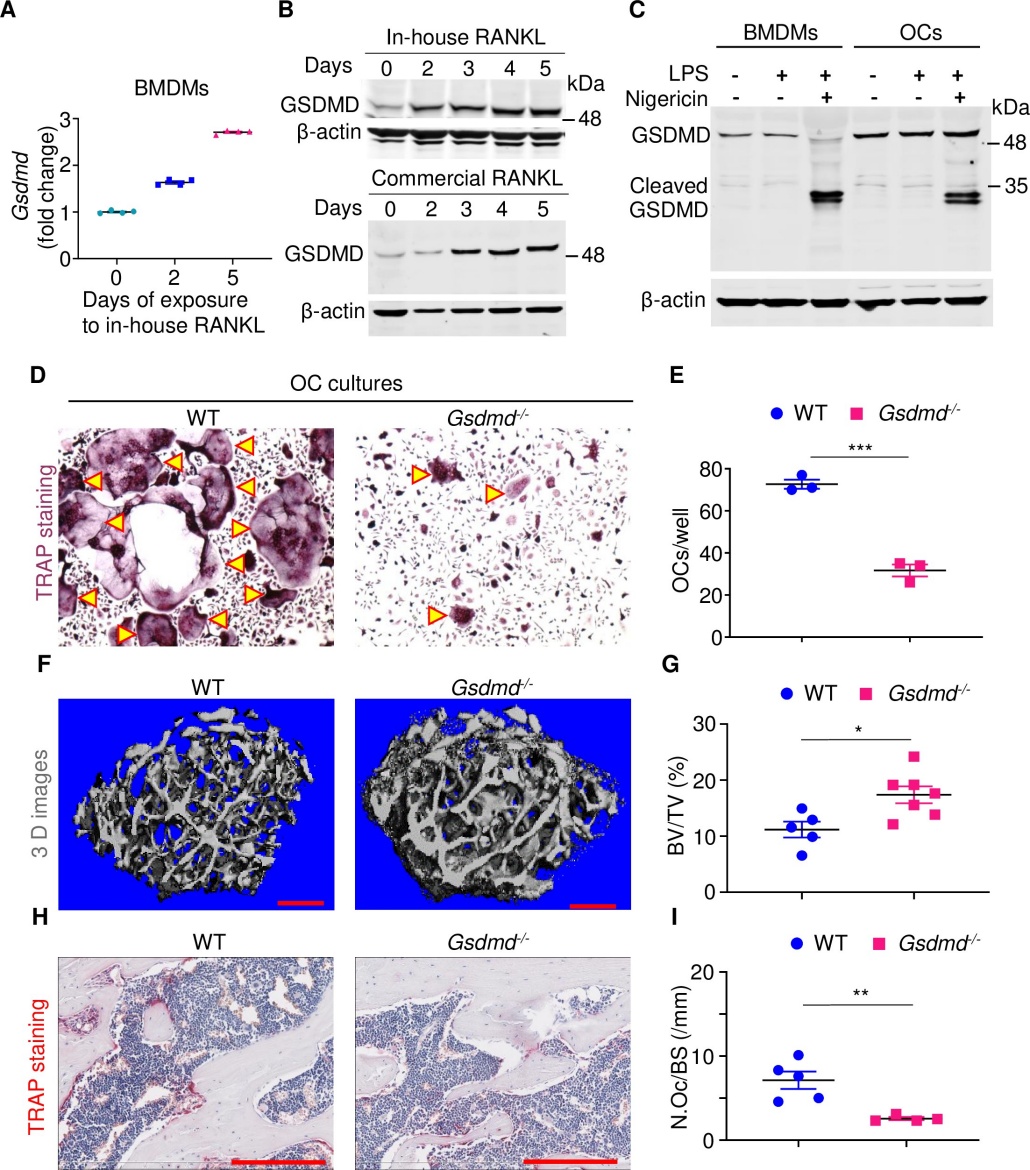
GSDMD exerts OC lineage autonomous actions. (A, B) BMDMs were treated with M-CSF and RANKL expressed in *Escherichia coli* (in-house RANKL) or commercial RANKL (to rule out potential effects caused by LPS contamination) for the indicated days. GSDMD expression was analyzed by qPCR (A) or immunoblotting (B). (C) BMDMs and OCs from BMDMs treated with M-CSF and RANKL for 4 days were exposed to 100 ng/ml LPS for 3 hours, then vehicle or 15 μM nigericin for 30 minutes. Cell lysates were analyzed by immunoblotting. (D, E) BMDMs from WT and *Gsdmd*^*-/*-^ mice were treated with M-CSF and RANKL for 4 days. Cultures were stained for TRAP activity. (D) Pictures of TRAP^+^ cells. (E) Counts of TRAP^+^ multinucleated cells (OCs, indicated by arrowheads). The femurs from 3-month-old WT and *Gsdmd*^*-/*-^ male mice were analyzed by μCT. (F) Cross sections of 3D reconstructions. (G) BV/TV. The femurs were also stained for TRAP activity. (H) TRAP^+^ cells (OCs), stained in red. (I) N.Oc/BS. Scale bar: 200 μm. The data underlying this figure may be found in S1 Data and S2 Data. Data are mean ± SEM. *P < 0.05; **P < 0.005; ***P < 0.0005. μCT, micro–computed tomography; BMDMs, bone marrow–derived macrophages; BV/TV, bone volume/total volume; GSDMD, gasdermin D; LPS, lipopolysaccharide; N.Oc/BS, OC number/bone surface; OC, osteoclast; qPCR, quantitative PCR; WT, wild-type.

The attenuated OC differentiation in *Gsdmd*^*-/-*^→*Gsdmd*^*-/-*^ mice may be the result of decreased secretion of inflammatory cytokines such as IL-1β, which promotes osteoclastogenesis through various mechanisms, including up-regulation of RANKL expression [36,49]. In addition, GSDMD may exert actions that are OC lineage autonomous. To test the latter scenario, we analyzed GSDMD expression during osteoclastogenesis. GSDMD mRNA and protein levels increased during in vitro OC differentiation of bone marrow–derived macrophages (BMDMs, Fig 4A–4C) and RAW 264.7 cells (S7A Fig) induced by RANKL. Although cleaved GSDMD was not detected during osteoclastogenesis, it was processed in OC cultures exposed to nigericin and lipopolysaccharide (LPS), though to a lesser extent compared to BMDMs (Fig 4C). Importantly, RANKL-driven OC formation was robust from WT BMDMs but impaired from *Gsdmd*^*-/-*^ cells (Fig 4D and 4E). More importantly, whereas baseline bone mass of non-littermate WT and *Gsdmd*^*-/-*^ male mice appeared comparable (Fig 2B and 2D), when littermates were used, bone mass was higher in *Gsdmd*^*-/-*^ mice compared to WT male controls (Fig 4F and 4G), a phenotype that correlated with OC parameters (Fig 4H and 4I). Notably, baseline indices of bone were comparable between WT and *Gsdmd*^*-/-*^ female mice (S3C and S3D Fig). Thus, whereas GSDMD is dispensable for bone homeostasis in female mice, it is crucial in physiological bone maintenance in male mice. In light of the OC-promoting actions of irradiation [50], our results suggest that irradiation may induce bone loss through nonautonomous and autonomous actions in immune cells and the OC cell lineage, respectively.

## Discussion

The AIM2 and NLRP3 inflammasomes are rapidly activated in response to perturbing signals, acting to clear the perceived danger and restore tissue homeostasis [51,52]. The failure of the organism to rapidly dampen danger signals and fine-tune inflammasome overreactions can be detrimental to health. These signals can be induced by ionizing radiation, as this procedure causes genomic instability and cytoplasmic accumulation of DNA, which can be recognized by AIM2 among other sensors [53]. This procedure also causes cell death, a rich source of the activators of the NLRP3 inflammasome such as ATP and uric acid [22,32,54,55]. Our results reinforce the view that radiation-inflammasome cascades are harmful, since the multiorgan damage induced by a high dose of radiation is profoundly attenuated when mice lack the inflammasome sensing components (NLRP3 or AIM2) in donor and recipient cells. Although a recent study suggests that focal irradiation promotes intestinal injury via the AIM2 inflammasome [18], here we show that TBI causes systemic tissue demise through the NLRP3 and AIM2 inflammasomes but not the NLRC4 inflammasome, actions that culminate in the activation of GSDMD. The residual tissue damage seen in some *Gsdmd-*deficient mice may be driven by GSDME, which is also expressed by hematopoietic cells, including macrophages, and mediates pyroptosis [56–60]. Another eloquent study shows that acute graft-versus-host disease (GvHD) is unaffected in *Nlrp3*^*-/-*^→WT mice, consistent with our results; however, this work conflicts with ours, as it finds that GvHD is significantly delayed in WT→*Nlrp3*^*-/-*^ mice [22]. Differences in the experimental designs and endpoint outcomes may account for the apparent discrepant observations. Our findings position GSDMD downstream of the NLRP3 and AIM2 inflammasomes in response to radiation-triggered signaling cascades. However, further studies are needed to determine whether or not NLRP3 and AIM2 are recruited to the same inflammasome complexes in irradiated cells as reported in murine cells exposed to cGAMP [61]. Since AIM2 senses DNA, it will also be interesting to determine the impact of deficiency in DNA repair on radiation-induced inflammasome activation. Poly(ADP-ribose) polymerases (PARPs), which are involved in DNA repair and affected by inflammasome signaling, are attractive candidates for such studies [62–66].

Serum levels of IL-1β correlate with the extent of bone loss in chimeric mice, findings that are consistent with the ability of this cytokine to regulate the expression of osteoclastogenic factors such as RANKL and to expand myeloid cell populations, some of which are OC precursors [36,67]. Serum levels of IL-1β in these mice also correlate with the magnitude of spleen enlargement, an outcome that is likely caused by the extensive EMH that occurs in this tissue as a homeostatic compensatory response to compromised hematopoiesis in the bone marrow. Consistent with skeletal outcomes, the increase in cytokine secretion and spleen weights are significantly reduced in chimeric mice fully lacking GSDMD. However, blood cell counts are unaffected in these mice compared to WT→*Gsdmd*^*-/-*^ mice, perhaps as the result of systemic inflammation in the latter mouse strain returning to baseline levels at the time of the sampling.

The increased levels of GSDMD during OC differentiation are unanticipated considering its pyroptotic actions. GSDMD is also unexpectedly not cleaved during osteoclastogenesis, yet its loss results in decreased OC differentiation both in vitro and in vivo. These results, in conjunction with those showing that LPS and nigericin induce the cleavage of this protein in OC cultures, rule out the scenario of defective inflammasome signaling in the OC lineage. However, the expression of GSDMD and maturation by terminally differentiated OCs still needs to be proven, given the cellular heterogeneity of OC cultures. Despite this knowledge gap, our findings revealing that GSDMD plays an important role in the differentiation of myeloid cells are novel because previous studies have focused on the function and regulation of this protein in inflammatory cells [25–27,68].

In summary, this study was driven by the hypothesis that the high sensitivity of the inflammasome pathways to homeostatic perturbations should, in theory, predispose these multifunctional protein platforms to aberrant activation by excessive ionizing radiation signals. Additionally, the inflammasomes should be activated not only in the irradiated host cells but also in the donor cells as they engraft in the damaged tissues. We have validated this new concept by demonstrating that suppression of inflammasome-GSDMD signaling in both donor and recipient cells is required to achieve efficacy upon exposure to a high dose of radiation.

## Materials and methods

### Ethics statement

All procedures were approved by the Institutional Animal Care and Use Committee (IACUC) of Washington University School of Medicine in St. Louis. All experiments were performed in accordance with the relevant guidelines and regulations described in the IACUC-approved protocol 19-0971.

### Mice

*Gsdmd* knockout (*Gsdmd*^−/−^) mice and *Nlrc4*^−/−^ mice were kindly provided by Dr. V. M. Dixit (Genentech, South San Francisco, CA, United States of America). *Aim2*^−/−^ mice were purchased from Jackson Laboratory (Sacramento, CA, USA). *Nlrp3*^−/−^ mice have been described previously [69]. All mice were on the C57BL6 background, and mouse genotyping was performed by PCR.

### BMT

Recipient mice were subjected to 9 Gy of TBI using X-RAD 320 Biological Irradiator (Precision X-Ray, North Branford, CT, USA). Bone marrow cells were flushed out from the femurs and tibias with phosphate buffered saline (PBS), filtered using 70-μm nylon mesh. Red blood cells were lysed, and 10^7^ bone marrow cells in 100 μl PBS were intravenously injected through the tail vein. Mice were supplied with water containing antibiotics (sulfamethoxazole and trimethoprim oral suspension [Ca# NDC 50383-824-16, AKORN, Lake Forest, IL, USA]).

### Flow cytometry

Bone marrow cells were flushed out from the femurs and tibias with PBS. Single cell suspensions at 1× 10^6^/200 μl were incubated with antibodies for 30 minutes at 4°C. The antibodies included CD3 PE-CY7 (Cat# 552774) and CD11b BB515 (Cat# 564454) from BD Biosciences (San Jose, CA, USA), B220 PerCP (Cat# 45-0452-82), CD115 PE (Cat# 12-1152-83), Gr-1 APC (Cat# 17-5931-82), and cKit e780 (Cat# 47-1172-82) from Thermo Fisher Scientific (Waltham, MA, USA). Flow cytometry analysis was performed using LSR Fortessa or FACS Canto II. Data were analyzed using FlowJo software (Tree Star).

### Bone microstructure and histomorphometry

The femurs were embedded in 2% agarose gel and scanned at 10 μm, 55 kVp, 145 μA, 8 W, 300-ms integration time using a μCT system (μCT 40; Scanco Medical AG, Zurich, Switzerland) as previously described [70,71]. The regions of interest (ROIs) were defined at 1 mm proximal to the end of the femoral growth plate.

For static histomorphometry, the femurs were fixed in 10% neutral buffered formalin for 16–24 hours, decalcified in 14% (w/v) EDTA (pH 7.2) for 10–14 days at room temperature, stored in 70% ethanol, paraffin-embedded, and sections at 5 μm. They were stained for tartrate-resistant acid phosphatase as previously described [72]. Images were acquired using a NanoZoomer 2.0 HT whole slide scanner (Hamamatsu Photonics, Hamamatsu City, Shizuoka, Japan) at 20× magnification. Bioquant Osteo software (v18.2.6; Bioquant Image Analysis Corp., Nashville, TN, USA) was used for image analysis. Static indices were measured in a 2‐mm ROI defined 100 μm from the end of the primary spongiosa.

For dynamic histomorphometry, mice were intraperitoneally injected with 10 mg/kg calcein green (Sigma-Aldrich, Cat# C0875, St. Louis, MO, USA) and 4 days later with 50 mg/kg alizarin red (Sigma-Aldrich, Cat# A3882). Mice were euthanized 2 days after the second injection. The left tibias and femurs were collected and fixed in 70% ethanol overnight, embedded in methyl methacrylate, and sectioned at 7–10 μm. Images were obtained using NanoZoomer. Measurements of dynamic bone histomorphometry were calculated from fluorochrome double labels at the endocortical surfaces as previously described [72].

For spleen histology, tissues were fixed in 10% formalin overnight, then embedded in paraffin followed by sectioning at 5 μm. After being mounted to positively charged glass slides, sections were dried and performed with hematoxylin-eosin (H&E) staining by standard methods.

### OC differentiation and TRAP staining

Mice were euthanized and bone marrow was flushed out from the tibias and femurs. BMDMs were obtained by culturing bone marrow cells in media containing 10% CMG, a source of M-CSF, for up to 5 days in a 10-cm dish as previously described [70,73]. After removing the nonadherent cells by vigorous washes with PBS, adherent cells were plated at 5 to 10 × 10^3^/well in a 96-well plate in culture media containing 2% CMG and 50–100 ng/ml RANKL. Cells were maintained at 37°C in a humidified atmosphere of 5% CO_2_, with media changed every other day. At the end of the culture period, the cells were rinsed with water and incubated with the TRAP staining solution (Sigma leukocyte acid phosphatase kit) at room temperature for 30 minutes. Multinucleated TRAP-positive cells with at least 3 nuclei were scored as OC under light microscopy.

### RNA isolation and RT-qPCR

RNA was extracted from cells by using RNeasy Plus Mini Kit (Qiagen). cDNA was prepared using High-Capacity cDNA Reverse Transcription Kits (Applied Biosystems). Gene expression was detected by qPCR using SYBR Green (Applied Biosystems) according to the manufacture. The data were analyzed using the ΔΔCT method normalizing against cyclophilin B.

### Western blot

BMDMs and OCs were primed with 100 ng/mL LPS for 3 hours, then treated with 15 μM nigericin for 30 minutes. To collect proteins, cells or tissues were lysed with RIPA buffer (50 mM Tris, 150 mM NaCl, 1 mM EDTA, 0.5% NaDOAc, 0.1% SDS, and 1.0% NP-40) plus Complete Protease Inhibitor Cocktail and phosphatase inhibitors (Roche, South San Francisco, CA, USA). Protein concentrations from cell lysates and tissue lysates were determined by Bio-Rad method. Proteins were separated by SDS-PAGE (12%) and transferred to PVDF membrane. Proteins were stained with antibodies against GSDMD (1:1,000, ab209845, Abcam), or β-actin (1:5,000, sc-47778, Santa Cruz Biotechnology, Dallas, TX, USA) overnight at 4°C, followed by a 1-hour incubation with secondary goat anti-rabbit IgG (1:5,000, A21109, Thermo Fisher Scientific, Waltham, MA, USA) or goat anti-mouse IgG (1:5,000, A21058, Thermo Fisher Scientific, Waltham, MA, USA), respectively. The signals were developed using Li-Cor Odyssey Infrared Imaging System (LI-COR Biosciences, Lincoln, NE, USA).

### Measurements of IL-1β and IL-18 levels

For IL-1β measurements in bone marrow, flushed bone marrow was centrifuged, and the supernatants were collected as described previously. IL-1β levels were measured and quantified using Luminex kits (Minneapolis, MN, USA).

### Statistical analysis

Statistical analysis was performed using Student *t* test, one-way ANOVA with Tukey’s multiple-comparisons test, or two-way ANOVA with Tukey’s multiple-comparisons test in GraphPad Prism7.

## Supporting information

S1 Fig Radiation causes bone injury. (A, B) Three-month-old WT (*tdT*^*-*^) male mice were subjected to 9-Gy TBI, then inoculated with bone marrow cells from *tdT*^*+*^ male mice to generate *tdT*^*+*^→*tdT*^*-*^ mice. Conversely, *tdT*^*-*^ cells were injected into irradiated *tdT*^*+*^ mice to obtain *tdT*^*-*^→*tdT*^*+*^ mice. Nonirradiated *tdT*^*+*^ mice and *tdT*^*-*^ male mice of the same age were used as positive and negative controls, respectively. Bone marrow cells were harvested 3 weeks later and analyzed by flow cytometry. (C-H) Three-month-old WT male mice left untreated or irradiated and transplanted with 10^7^ bone marrow cells (WT→WT mice) were labeled with calcein green and alizarin red. The femurs were analyzed by μCT analysis. (C) Cross sections of 3D reconstructions. (D) BV/TV. The femurs were also stained for TRAP activity. (E) TRAP^+^ cells (OCs), stained in red. (F) N.Oc/BS. The tibias were also analyzed by histology. (G) Pictures of double-labeled bone surfaces. (H) MAR. The numerical values underlying S1B, D, F, H Fig can be found in S1 Data. Data are mean ± SEM. **P < 0.005; ***P < 0.0005, ****P < 0.0001. μCT, micro–computed tomography; BV/TV, bone volume/total volume; MAR, mineral apposition rate; N.Oc/BS, OC number/bone surface; OC, osteoclast; TBI, total body irradiation; tdT, tdTomato; WT, wild-type.
(TIF)

S2 Fig Radiation damages bones through the NLRP3 and AIM2 inflammasomes but not the NLRC4 inflammasome. Three-month-old WT and *Nlrp3*^*-/-*^ male mice were left untreated or subjected to 9-Gy TBI. Irradiated mice were transplanted with 10^7^ bone marrow cells from 3-month-old WT or *Nlrp3*^*-/-*^ male mice to generate *Nlrp3*^*-/-*^→WT, WT→WT, WT→*Nlrp3*^*-/-*^, *Nlrp3*^*-/-*^→*Nlrp3*^*-/-*^, WT→*Aim2*^*-/-*^, *Aim2*^*-/-*^→*Aim2*^*-/-*^, WT→*Nlrc4*^*-/-*^, and *Nlrc4*^*-/-*^→*Nlrc4*^*-/-*^ mice. The femurs were analyzed by μCT. (A, C, E, G) vBMD. The femurs were also stained for TRAP activity. (B, D, F, H) Oc.S/BS. Scale bar: 200 μm. The numerical values underlying S2A–S2H Fig can be found in S1 Data. Data are mean ± SEM. *P < 0.05, **P < 0.005, ***P < 0.0005. μCT, micro–computed tomography; ns, not significant; Oc.S/BS, OC surface/bone surface; TBI, total body irradiation; vBMD, volumetric bone mineral density; WT, wild-type
(TIF)

S3 Fig Radiation damages bones through GSDMD. Three-month-old WT and *Gsdmd*^*-/-*^ female mice (A-D) and male mice (E) were left untreated or subjected to 9-Gy TBI. Irradiated mice were transplanted with 10^7^ bone marrow cells from 3-month-old WT or null mice of the corresponding sex to generate WT→WT, *Gsdmd*^*-/-*^→WT, WT→*Gsdmd*^*-/-*^ and *Gsdmd*^*-/-*^→*Gsdmd*^*-/-*^ mice. The femurs were analyzed by μCT. (A, C) Cross sections of 3D reconstructions. (B, D) BV/TV. The femurs were also stained for TRAP activity. (E) Oc.S/BS. The numerical values underlying S3B, D, E Fig can be found in S1 Data. Data are mean ± SEM. ***P < 0.0005; ****P < 0.0001. Scale bar: 200 μm. μCT, micro–computed tomography; BV/TV, bone volume/total volume; GSDMD, gasdermin D; ns, not significant; Oc.S/BS, OC surface/bone surface; TBI, total body irradiation; WT, wild-type.
(TIF)

S4 Fig Gating strategy for the flow cytometry data shown in Fig 2G. (TIF)

S5 Fig Radiation damages the spleen through the NLRP3 and AIM2 inflammasomes but not the NLRC4 inflammasome. Three-month-old *Nlrp3*^*-/-*^, *Aim2*^*-/-*^, or *Nlrc4*^*-/-*^ male mice were left untreated or subjected to 9-Gy TBI. Irradiated mice were transplanted with 10^7^ bone marrow cells from 3-month-old male mice to generate WT→*Nlrp3*^*-/-*^ and *Nlrp3*^*-/-*^→*Nlrp3*^*-/-*^ mice (A), WT→*Aim2*^*-/-*^ and *Aim2*^*-/-*^→*Aim2*^*-/-*^ mice (B), and WT→*Nlrc4*^*-/-*^ and *Nlrc4*^*-/-*^→*Nlrc4*^*-/-*^ mice (C). The spleen was analyzed 3 weeks later; the weight was normalized to the body weight. The numerical values underlying S5A–C Fig can be found in S1 Data. Data are mean ± SEM. *P < 0.05; **P < 0.005; ***P < 0.0005; ****P < 0.0001. ns, not significant; TBI, total body irradiation; WT, wild-type.
(TIF)

S6 Fig Radiation-induced tissue injury correlates with the extent of GSDMD activation. (A, B, E-J) Three-month-old WT mice or *Gsdmd*^*-/-*^ male mice were left untreated or subjected to 9-Gy TBI. Irradiated mice were transplanted with 10^7^ bone marrow cells from 3-month-old WT or *Gsdmd*^*-/-*^ male mice to generate WT→WT, *Gsdmd*^*-/-*^→*Gsdmd*^*-/-*^, and WT→*Gsdmd*^*-/-*^ mice. (C, D) Three-month-old WT male mice were left untreated or subjected to 9-Gy TBI; irradiated mice were not transplanted with bone marrow cells. Samples were collected 2 or 7 days post TBI/BMT (A, B); 3 weeks post-TBI/BMT (E-J); and 1, 2, or 3 days after TBI (C, D); and analyzed alongside control lysates by immunoblotting (1–2 mice/group) or flow cytometry. The data underlying this figure may be found in S1 Data and S2 Data. Data are mean ± SEM. **P < 0.005; ****P < 0.0001. BMT, bone marrow transplantation; GSDMD, gasdermin D; ns, not significant; TBI, total body irradiation; WT, wild-type.
(TIF)

S7 Fig GSDMD expression increases during OC differentiation. (A) RAW 264.7 cells were treated with RANKL for the indicated days. GSDMD expression was analyzed by qPCR. The numerical values underlying S7A Fig can be found in S1 Data. (B) Data are from http://biogps.org/#goto=genereport&id=69146. GSDMD, gasdermin D; OC, osteoclast; qPCR, quantitative PCR.
(TIF)

S1 Data This spreadsheet contains raw data used to plot the graphs Fig 1B, 1D, 1F, 11H, 1J, 1L and 1N; Fig 2B, 2D, 2F, 2G and 2I; Fig 3E and 3F; Fig 4A, 4E, 4G and 4I; S1B, S1D, S1F and S1H Fig; S2A–S2H Fig; S3B, S3D, and S3E Fig; S5A–S5C Fig; S6E–S6J Fig; and S7A Fig. (XLSX)

S2 Data This file contains the original images supporting the blots for Fig 3A–3D, Fig 4B and 4C, S6A–S6D Fig. (PPTX)

## Abbreviations

μCT: micro–computed tomography
ACT: adoptive cell transfer
BMDM: bone marrow–derived macrophage
BMT: bone marrow transplantation
BV/TV: bone volume/total volume
DAMP: danger-associated molecular pattern
EMH: extramedullary hematopoiesis
GSDMD: gasdermin D
GSDMD^Ct^: C-terminal GSDMD
GSDMD^Nt^: N-terminal GSDMD
GvHD: graft-versus-host disease
HMGB1: high mobility group box 1
HSC: hematopoietic stem cell
LPS: lipopolysaccharide
ns: not significant
OC: osteoclast
PARP: poly(ADP-ribose) polymerase
PMN: polymorphonuclear cell
pro-IL-1β: pro-interleukin-1β
qPCR: quantitative PCR
TBI: total body irradiation
tdT: tdTomato
WT: wild-type
