# Supplementary figures and images for: Radiation causes tissue damage by dysregulating inflammasome–gasdermin D signaling in both host and transplanted cells
Source: PLoS Biol. 2020 Aug 6;18(8):e3000807. doi: 10.1371/journal.pbio.3000807 (PMC7446913; doi:10.1371/journal.pbio.3000807)

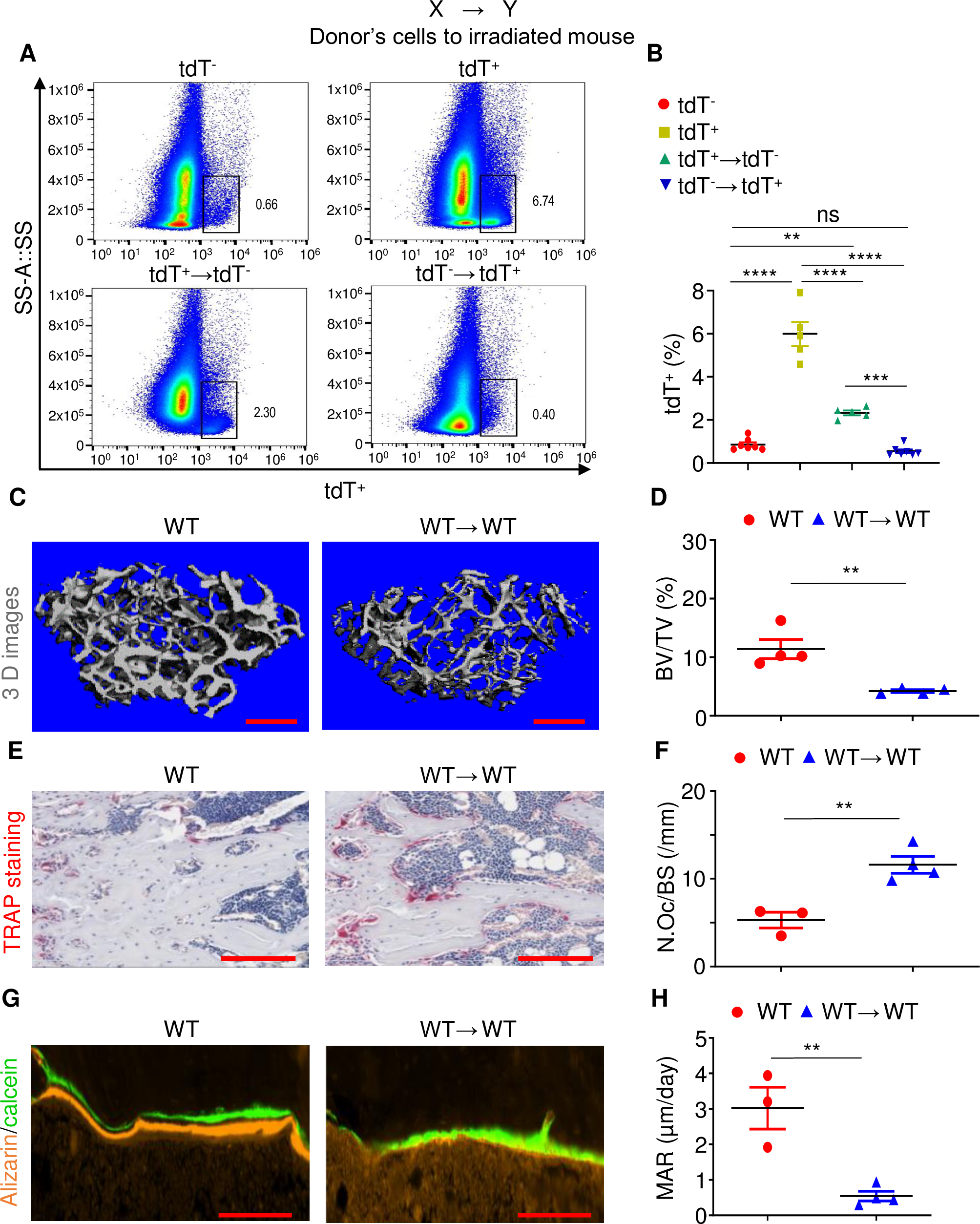

Supplement: S1 Fig — (A, B) Three-month-old WT (tdT-) male mice were subjected to 9-Gy TBI, then inoculated with bone marrow cells from tdT+ male mice to generate tdT+→tdT- mice. Conversely, tdT- cells were injected into irradiated tdT+ mice to obtain tdT-→tdT+ mice. Nonirradiated tdT+ mice and tdT- male mice of the same age were used as positive and negative controls, respectively. Bone marrow cells were harvested 3 weeks later and analyzed by flow cytometry. (C-H) Three-month-old WT male mice left untreated or irradiated and transplanted with 107 bone marrow cells (WT→WT mice) were labeled with calcein green and alizarin red. The femurs were analyzed by μCT analysis. (C) Cross sections of 3D reconstructions. (D) BV/TV. The femurs were also stained for TRAP activity. (E) TRAP+ cells (OCs), stained in red. (F) N.Oc/BS. The tibias were also analyzed by histology. (G) Pictures of double-labeled bone surfaces. (H) MAR. The numerical values underlying S1B, D, F, H Fig can be found in S1 Data. Data are mean ± SEM. **P < 0.005; ***P < 0.0005, ****P < 0.0001. μCT, micro–computed tomography; BV/TV, bone volume/total volume; MAR, mineral apposition rate; N.Oc/BS, OC number/bone surface; OC, osteoclast; TBI, total body irradiation; tdT, tdTomato; WT, wild-type. (TIF) [file pbio.3000807.s001.tif]

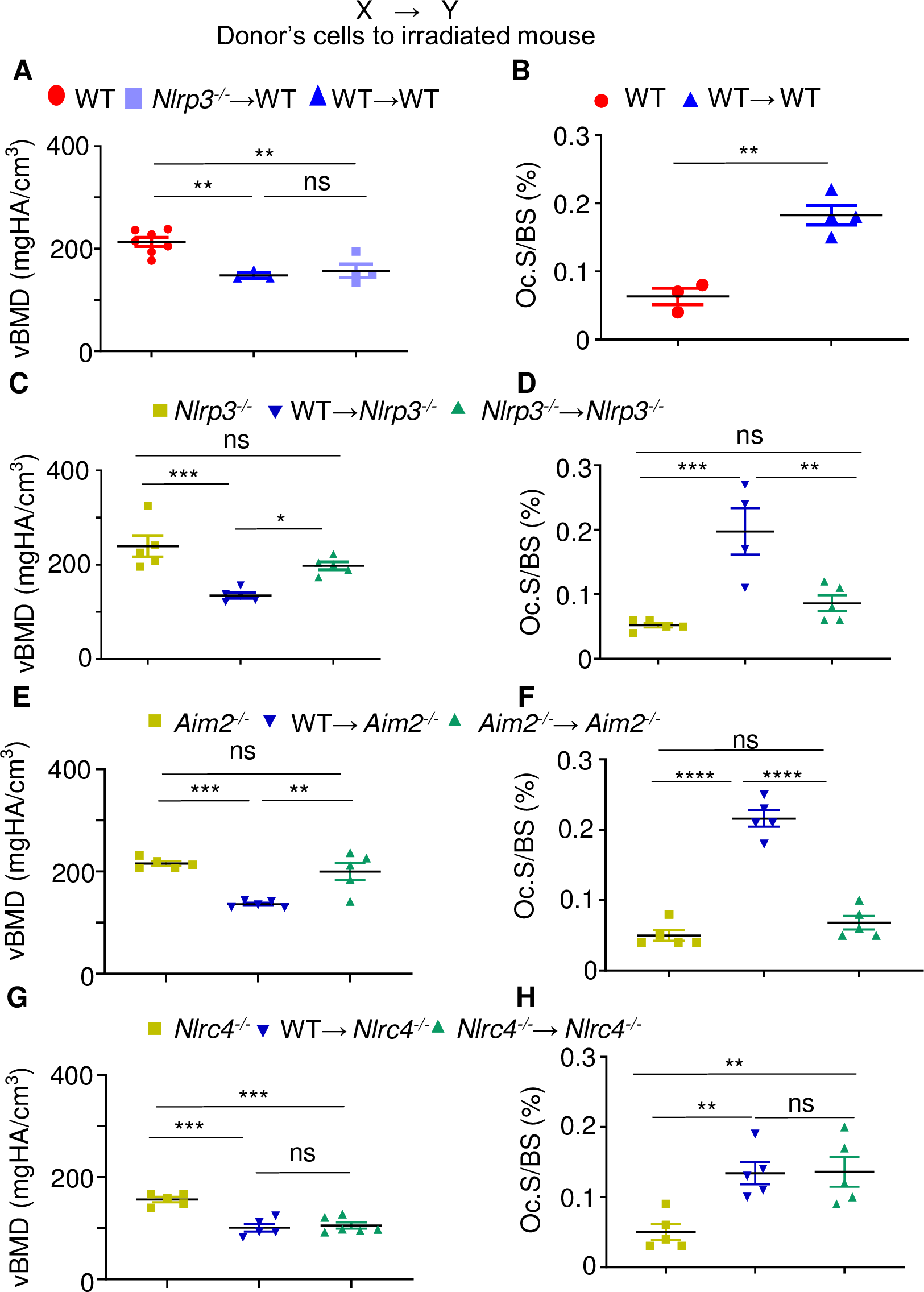

Supplement: S2 Fig — Three-month-old WT and Nlrp3-/- male mice were left untreated or subjected to 9-Gy TBI. Irradiated mice were transplanted with 107 bone marrow cells from 3-month-old WT or Nlrp3-/- male mice to generate Nlrp3-/-→WT, WT→WT, WT→Nlrp3-/-, Nlrp3-/-→Nlrp3-/-, WT→Aim2-/-, Aim2-/-→Aim2-/-, WT→Nlrc4-/-, and Nlrc4-/-→Nlrc4-/- mice. The femurs were analyzed by μCT. (A, C, E, G) vBMD. The femurs were also stained for TRAP activity. (B, D, F, H) Oc.S/BS. Scale bar: 200 μm. The numerical values underlying S2A–S2H Fig can be found in S1 Data. Data are mean ± SEM. *P < 0.05, **P < 0.005, ***P < 0.0005. μCT, micro–computed tomography; ns, not significant; Oc.S/BS, OC surface/bone surface; TBI, total body irradiation; vBMD, volumetric bone mineral density; WT, wild-type (TIF) [file pbio.3000807.s002.tif]

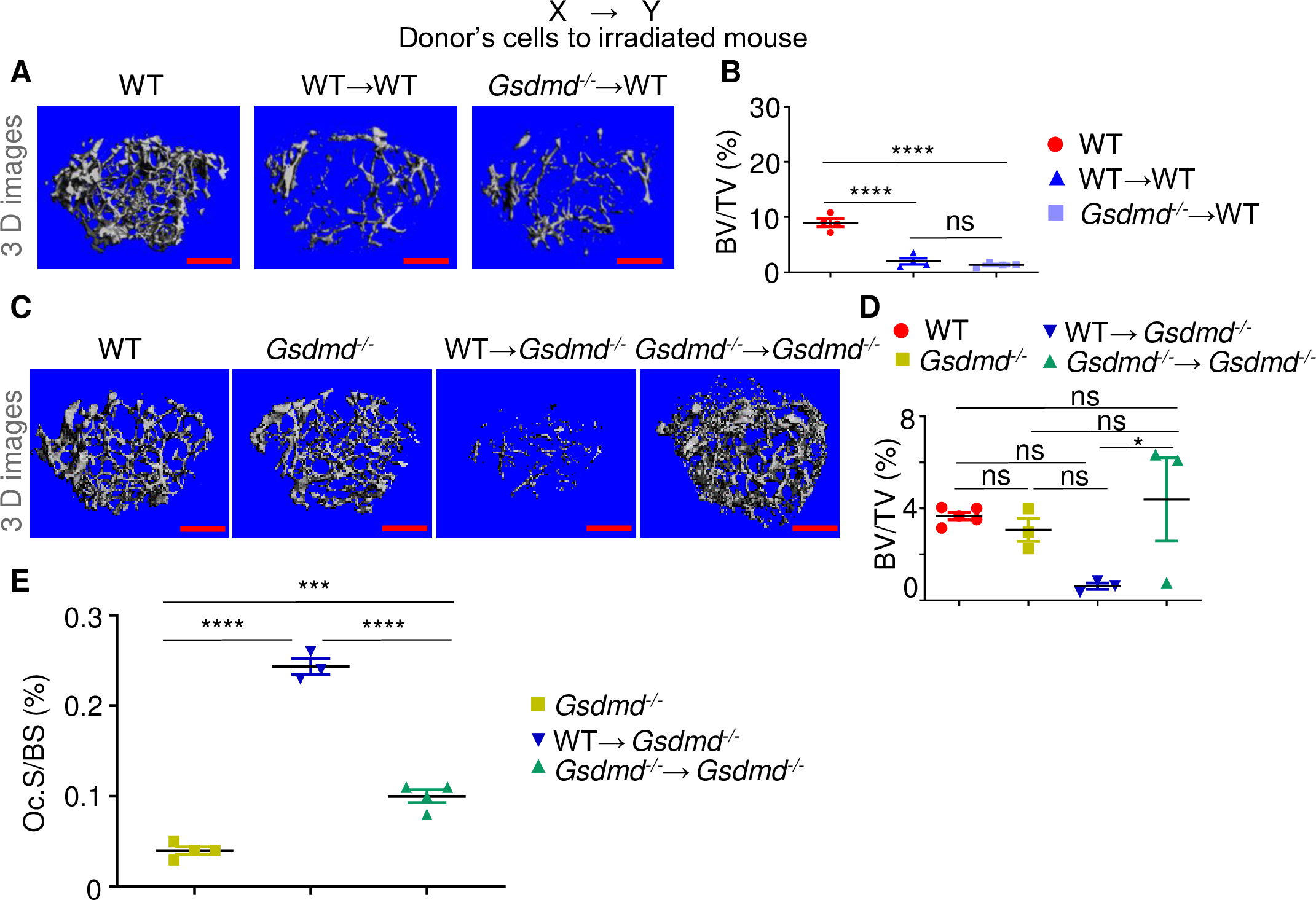

Supplement: S3 Fig — Three-month-old WT and Gsdmd-/- female mice (A-D) and male mice (E) were left untreated or subjected to 9-Gy TBI. Irradiated mice were transplanted with 107 bone marrow cells from 3-month-old WT or null mice of the corresponding sex to generate WT→WT, Gsdmd-/-→WT, WT→Gsdmd-/- and Gsdmd-/-→Gsdmd-/- mice. The femurs were analyzed by μCT. (A, C) Cross sections of 3D reconstructions. (B, D) BV/TV. The femurs were also stained for TRAP activity. (E) Oc.S/BS. The numerical values underlying S3B, D, E Fig can be found in S1 Data. Data are mean ± SEM. ***P < 0.0005; ****P < 0.0001. Scale bar: 200 μm. μCT, micro–computed tomography; BV/TV, bone volume/total volume; GSDMD, gasdermin D; ns, not significant; Oc.S/BS, OC surface/bone surface; TBI, total body irradiation; WT, wild-type. (TIF) [file pbio.3000807.s003.tif]

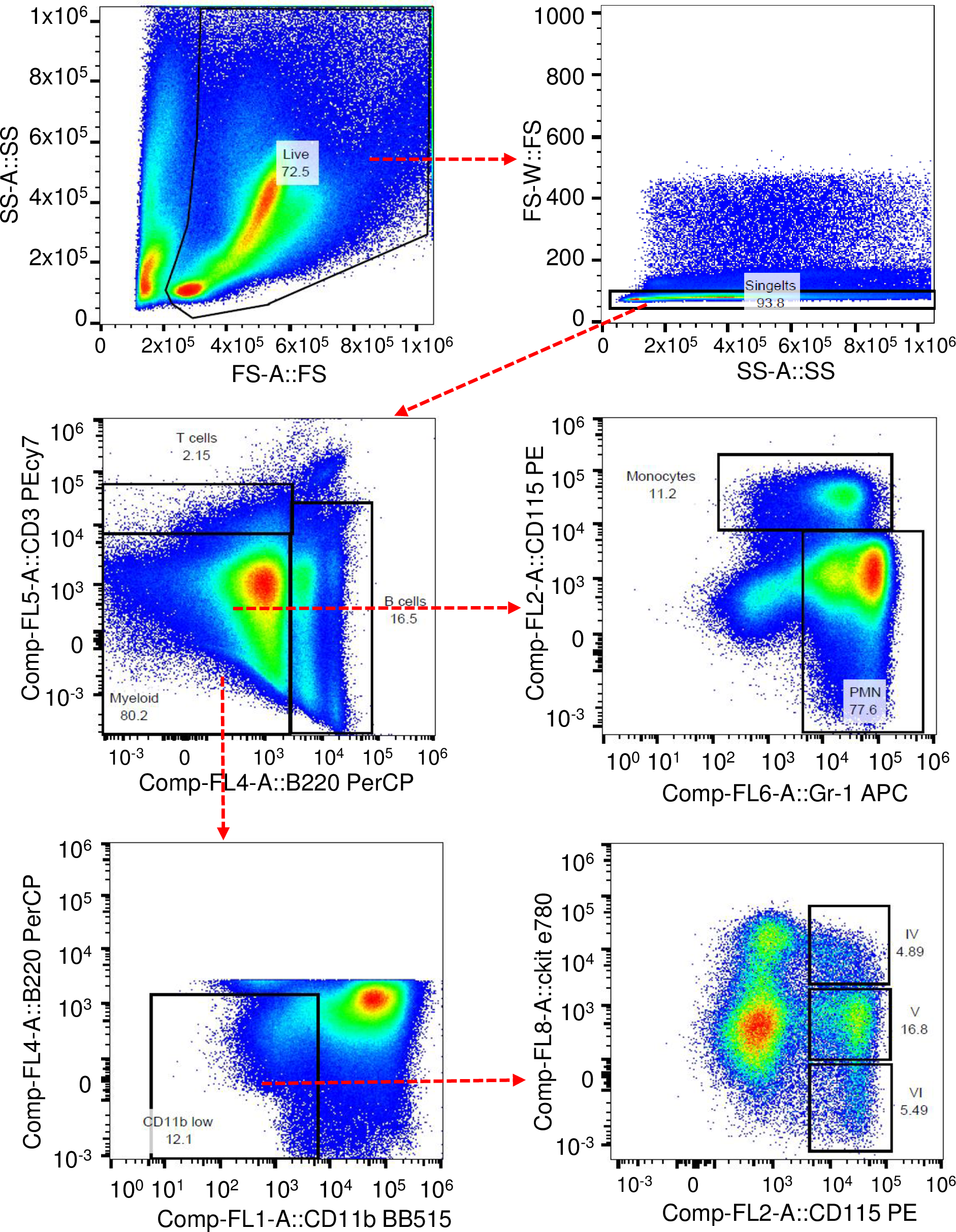

Supplement: S4 Fig — (TIF) [file pbio.3000807.s004.tif]

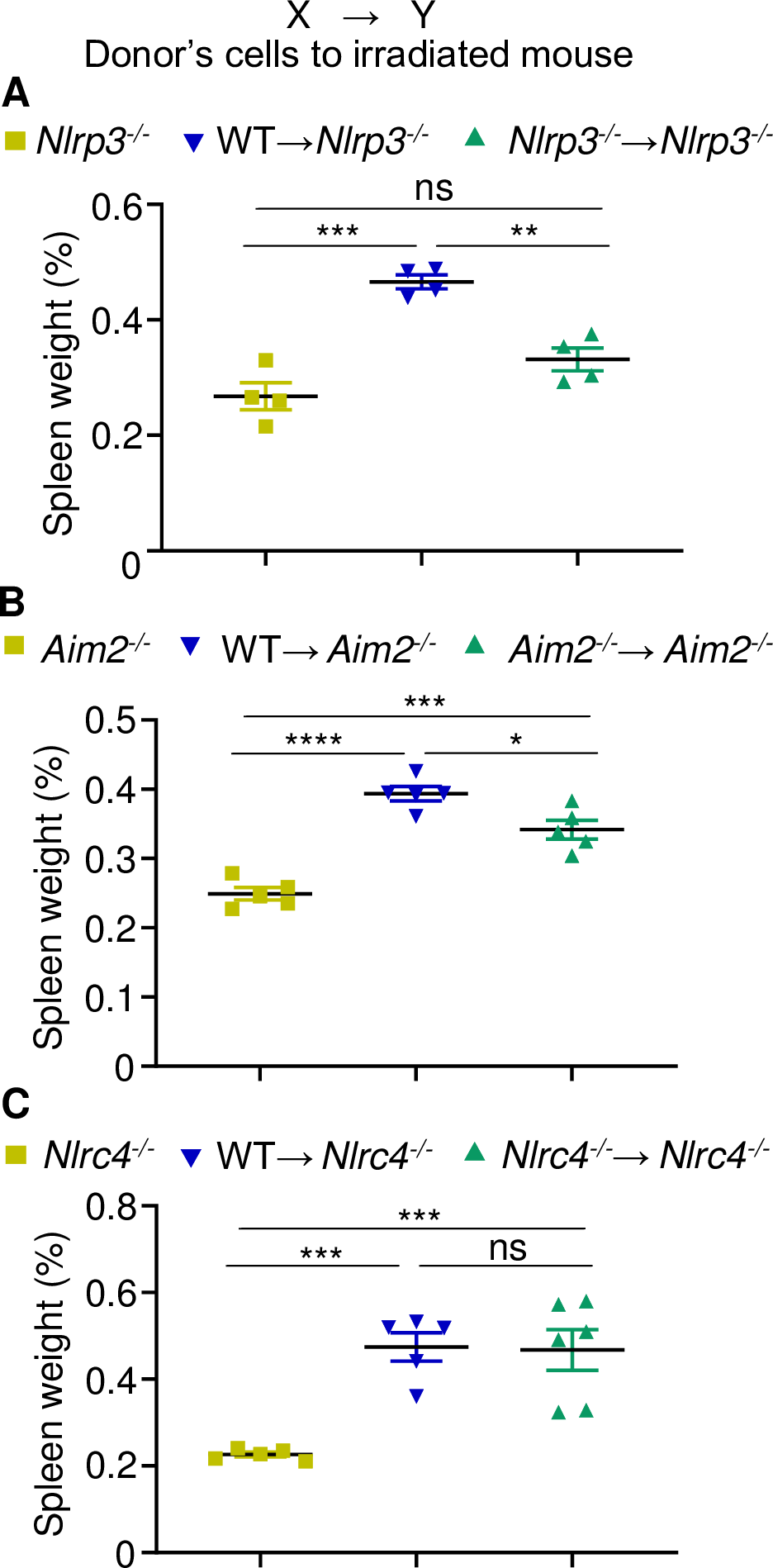

Supplement: S5 Fig — Three-month-old Nlrp3-/-, Aim2-/-, or Nlrc4-/- male mice were left untreated or subjected to 9-Gy TBI. Irradiated mice were transplanted with 107 bone marrow cells from 3-month-old male mice to generate WT→Nlrp3-/- and Nlrp3-/-→Nlrp3-/- mice (A), WT→Aim2-/- and Aim2-/-→Aim2-/- mice (B), and WT→Nlrc4-/- and Nlrc4-/-→Nlrc4-/- mice (C). The spleen was analyzed 3 weeks later; the weight was normalized to the body weight. The numerical values underlying S5A–C Fig can be found in S1 Data. Data are mean ± SEM. *P < 0.05; **P < 0.005; ***P < 0.0005; ****P < 0.0001. ns, not significant; TBI, total body irradiation; WT, wild-type. (TIF) [file pbio.3000807.s005.tif]

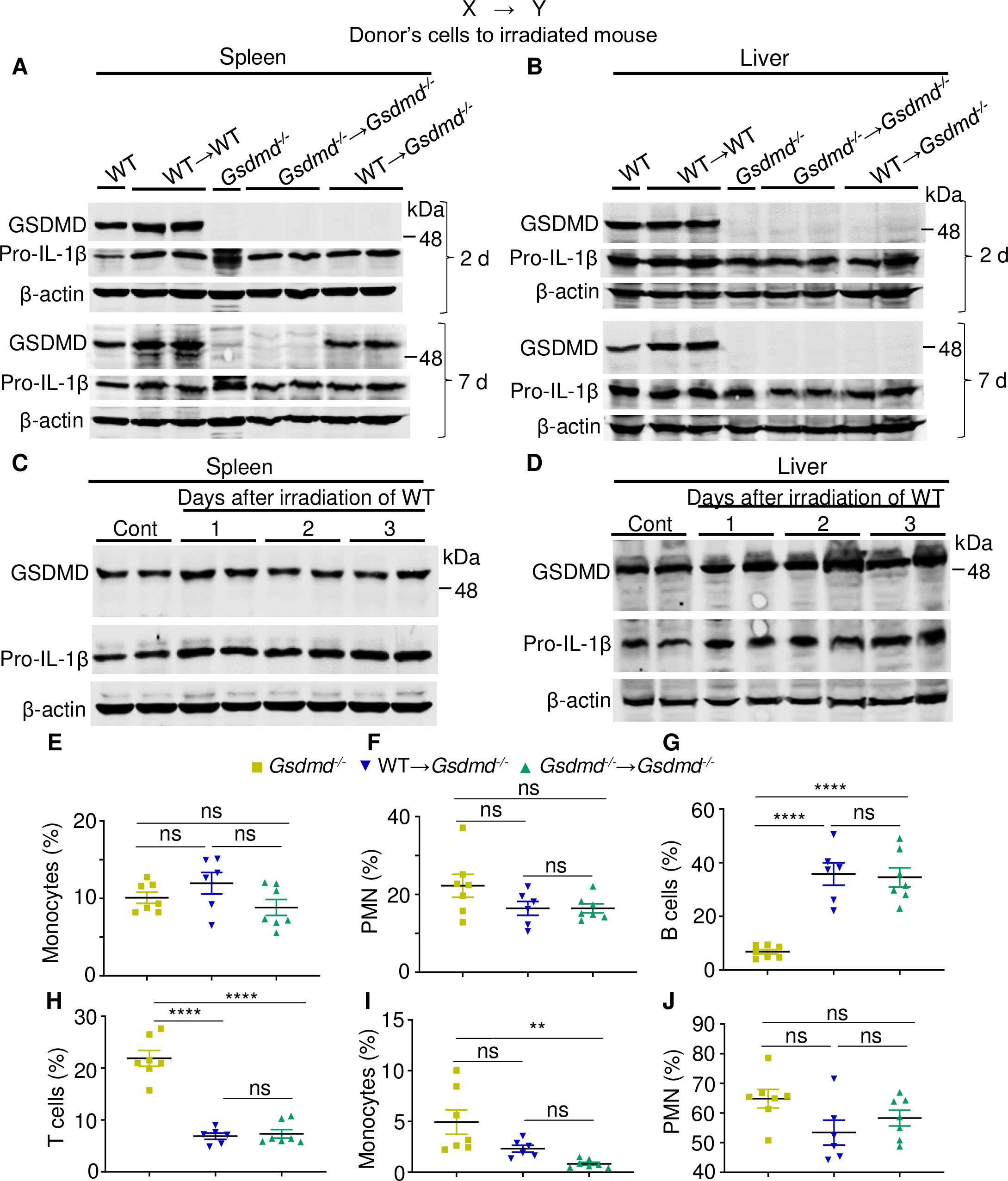

Supplement: S6 Fig — (A, B, E-J) Three-month-old WT mice or Gsdmd-/- male mice were left untreated or subjected to 9-Gy TBI. Irradiated mice were transplanted with 107 bone marrow cells from 3-month-old WT or Gsdmd-/- male mice to generate WT→WT, Gsdmd-/-→Gsdmd-/-, and WT→Gsdmd-/- mice. (C, D) Three-month-old WT male mice were left untreated or subjected to 9-Gy TBI; irradiated mice were not transplanted with bone marrow cells. Samples were collected 2 or 7 days post TBI/BMT (A, B); 3 weeks post-TBI/BMT (E-J); and 1, 2, or 3 days after TBI (C, D); and analyzed alongside control lysates by immunoblotting (1–2 mice/group) or flow cytometry. The data underlying this figure may be found in S1 Data and S2 Data. Data are mean ± SEM. **P < 0.005; ****P < 0.0001. BMT, bone marrow transplantation; GSDMD, gasdermin D; ns, not significant; TBI, total body irradiation; WT, wild-type. (TIF) [file pbio.3000807.s006.tif]

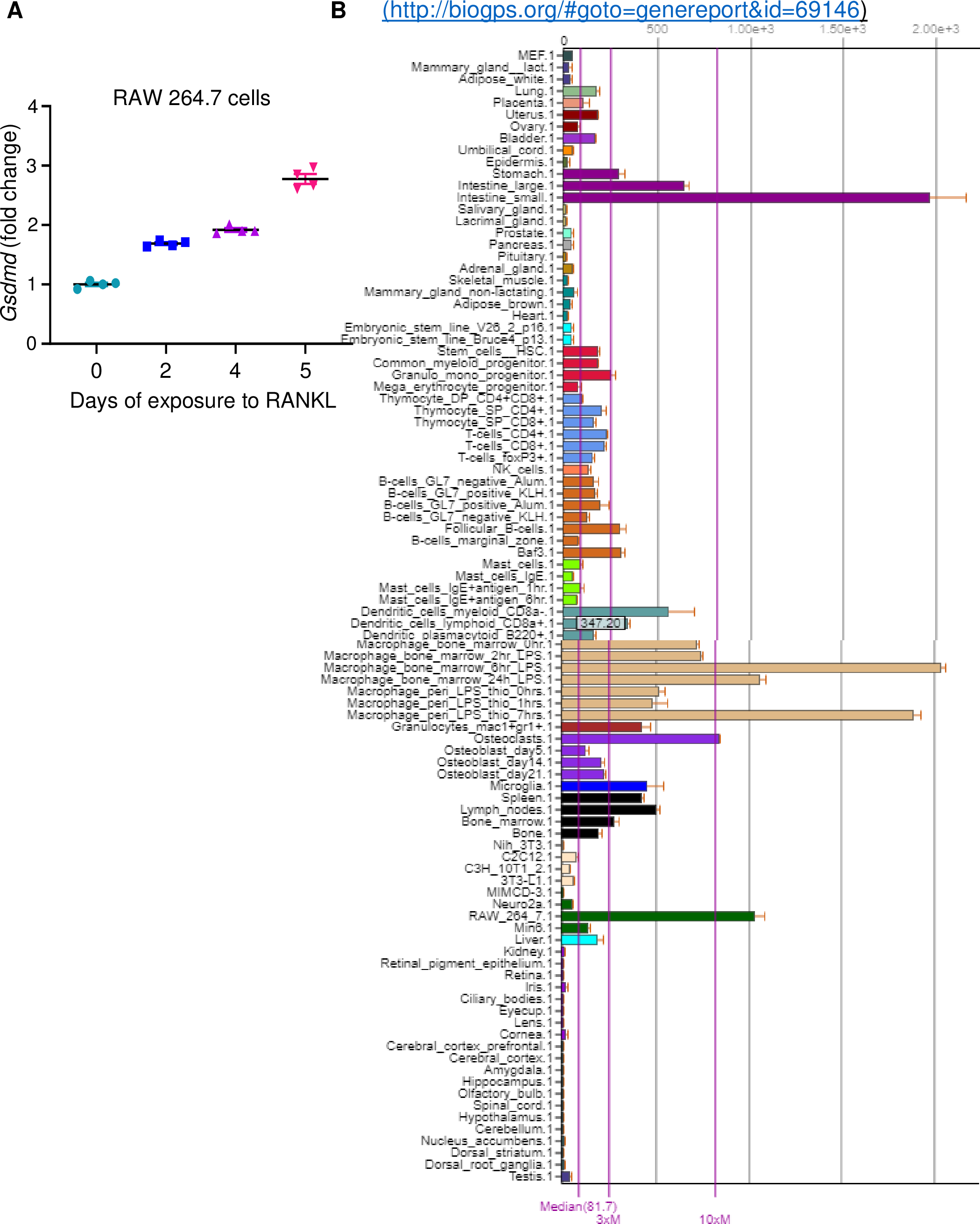

Supplement: S7 Fig — (A) RAW 264.7 cells were treated with RANKL for the indicated days. GSDMD expression was analyzed by qPCR. The numerical values underlying S7A Fig can be found in S1 Data. (B) Data are from http://biogps.org/#goto=genereport&id=69146. GSDMD, gasdermin D; OC, osteoclast; qPCR, quantitative PCR. (TIF) [file pbio.3000807.s007.tif]
