# Supplementary material for: Radiation causes tissue damage by dysregulating inflammasome–gasdermin D signaling in both host and transplanted cells
Source: PLoS Biol. 2020 Aug 6;18(8):e3000807. doi: 10.1371/journal.pbio.3000807 (PMC7446913; doi:10.1371/journal.pbio.3000807)
Supplement: S2 Data — (PPTX) [file pbio.3000807.s009.pptx]

## Slide 1
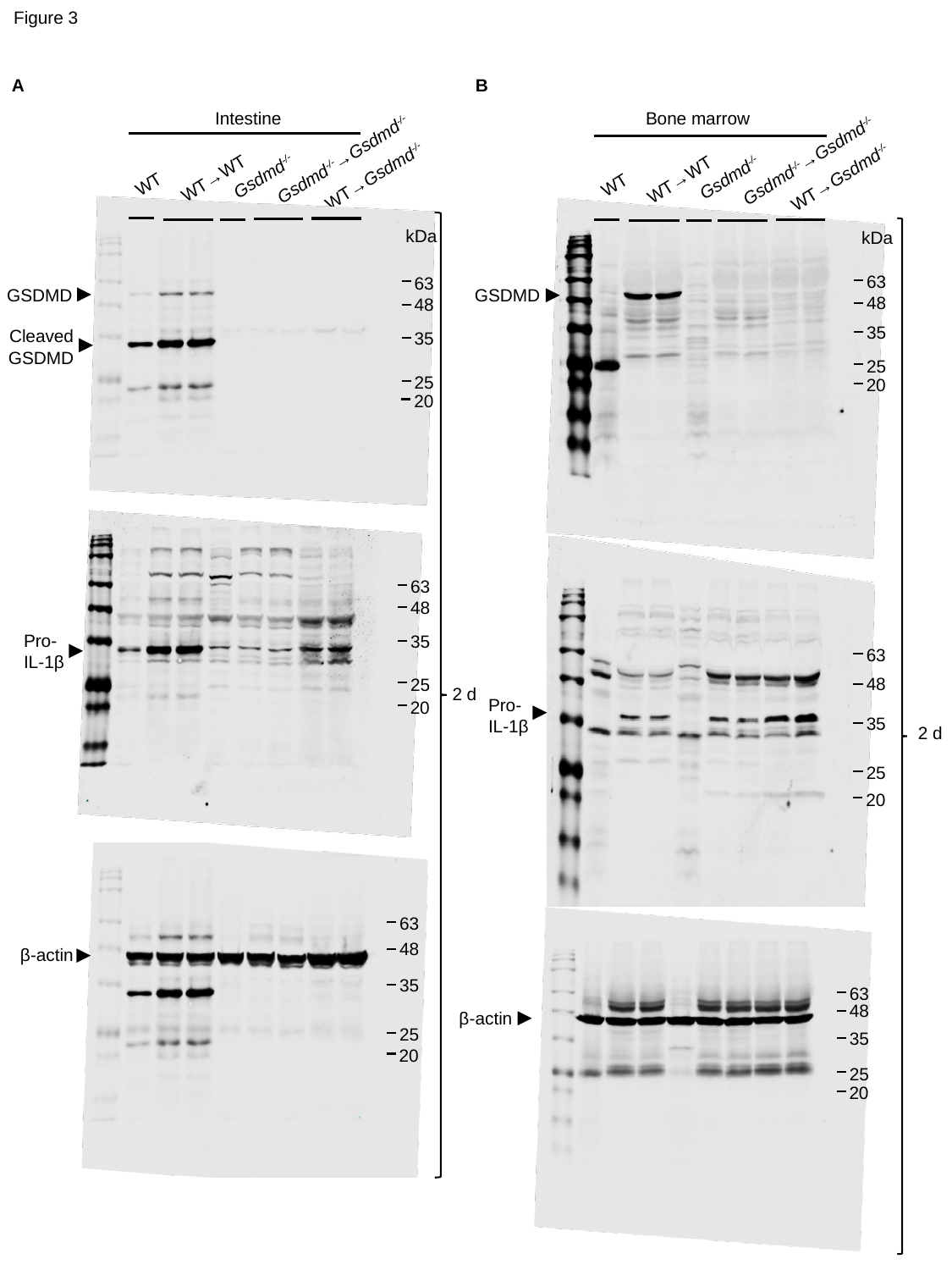

Figure 3
A B
Intestine Bone marrow
Gsdmd-/-→Gsdmd-/-
Gsdmd-/-→Gsdmd-/-
WT→Gsdmd-/-
WT→Gsdmd-/-
WT→WT
Gsdmd-/-
WT→WT
Gsdmd-/-
WT
WT
 kDa
 kDa
63
63
GSDMD
GSDMD
48
48
35
Cleaved
35
GSDMD
25
25
20
20
63
48
Pro-IL-1β
35
63
48
25
2 d
Pro-IL-1β
20
35
2 d
25
20
63
48
β-actin
35
63
48
β-actin
25
35
20
25
20

## Slide 2
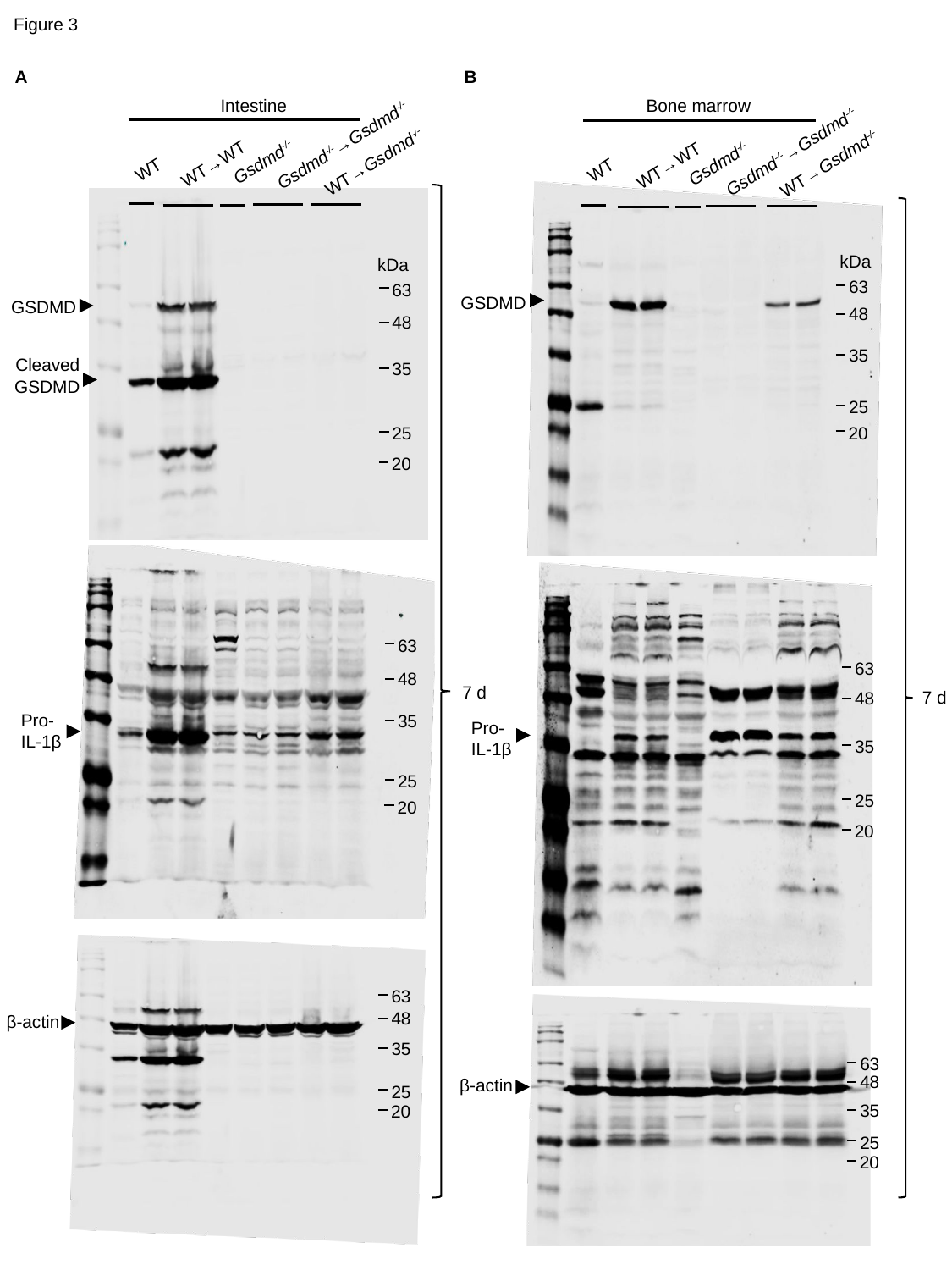

Figure 3
A B
Intestine Bone marrow
Gsdmd-/-→Gsdmd-/-
Gsdmd-/-→Gsdmd-/-
WT→Gsdmd-/-
WT→Gsdmd-/-
WT→WT
Gsdmd-/-
WT→WT
Gsdmd-/-
WT
WT
 kDa
 kDa
63
63
GSDMD
GSDMD
48
48
35
Cleaved
35
GSDMD
25
20
25
20
63
63
48
7 d
7 d
48
Pro-IL-1β
35
Pro-IL-1β
35
25
25
20
20
63
48
β-actin
35
63
48
β-actin
25
35
20
25
20

## Slide 3
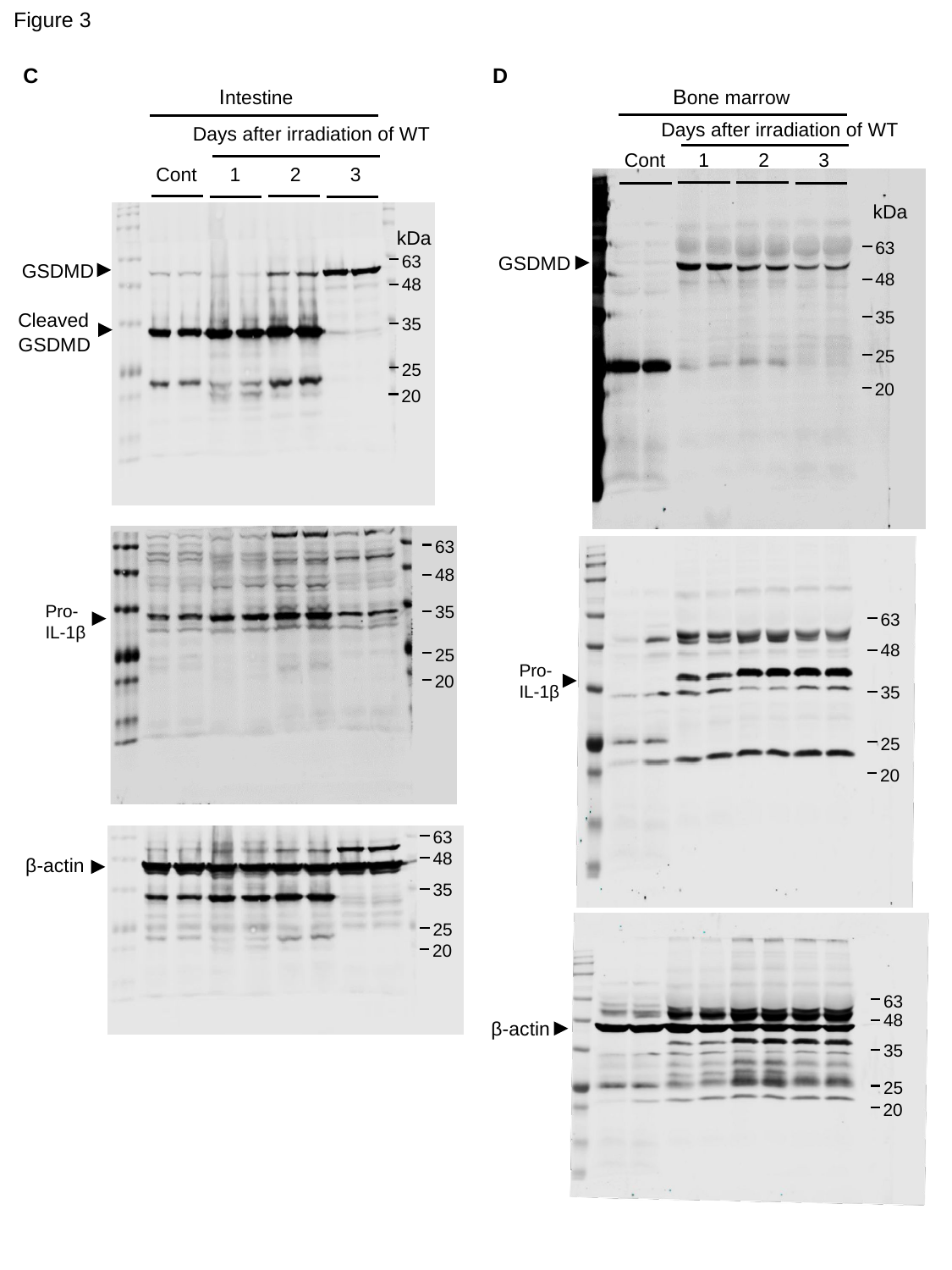

Figure 3
C D
Intestine Bone marrow
Days after irradiation of WT
Days after irradiation of WT
Cont 1 2 3
Cont 1 2 3
 kDa
 kDa
63
63
GSDMD
GSDMD
48
48
35
Cleaved
35
GSDMD
25
25
20
20
63
48
Pro-IL-1β
35
63
48
25
Pro-IL-1β
20
35
25
20
63
48
β-actin
35
25
20
63
48
β-actin
35
25
20

## Slide 4
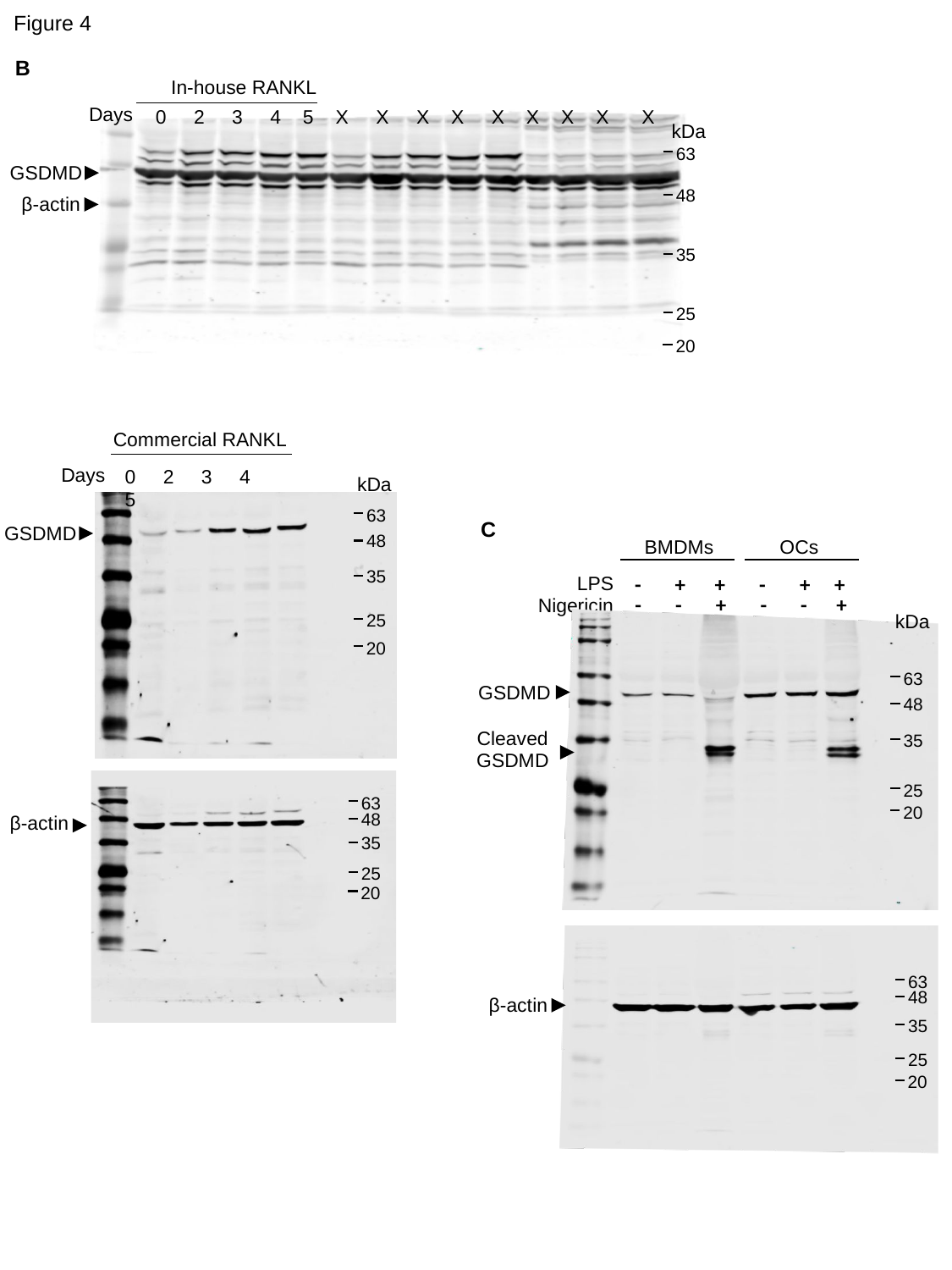

Figure 4
B
In-house RANKL
Days
0 2 3 4 5 X X X X X X X X X
 kDa
63
GSDMD
48
β-actin
35
25
20
Commercial RANKL
Days
0 2 3 4 5
 kDa
63
C
GSDMD
48
BMDMs
OCs
35
LPS
- + + - + +
- - + - - +
Nigericin
25
 kDa
20
63
GSDMD
48
Cleaved
35
GSDMD
25
63
20
48
β-actin
35
25
20
63
48
β-actin
35
25
20

## Slide 5
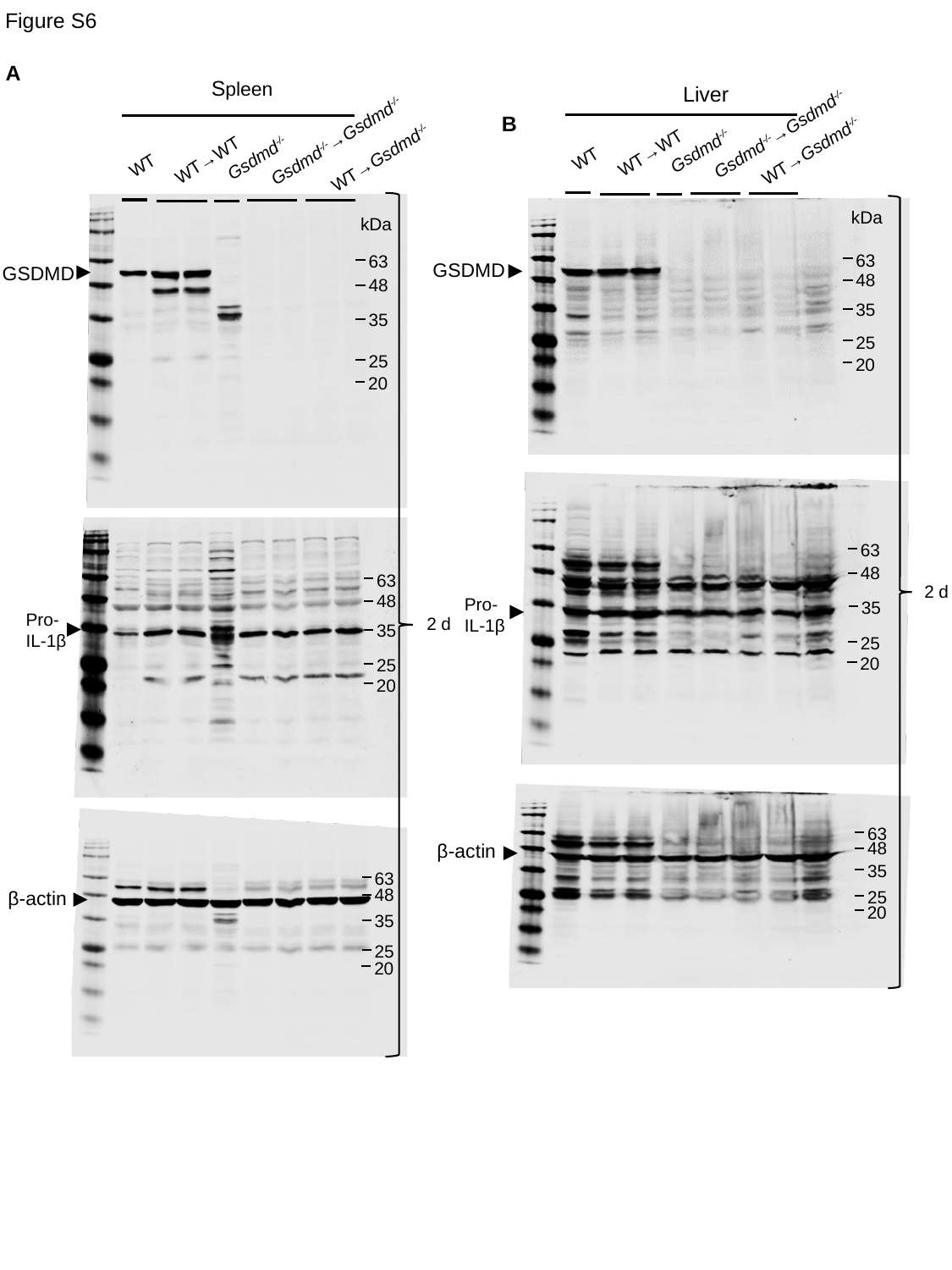

Figure S6
A
Spleen
Liver
B
Gsdmd-/-→Gsdmd-/-
Gsdmd-/-→Gsdmd-/-
WT→Gsdmd-/-
WT→WT
Gsdmd-/-
WT→Gsdmd-/-
WT→WT
Gsdmd-/-
WT
WT
 kDa
 kDa
63
63
GSDMD
GSDMD
48
48
35
35
25
25
20
20
63
48
63
2 d
48
Pro-IL-1β
35
Pro-IL-1β
2 d
35
25
20
25
20
63
48
β-actin
35
63
48
β-actin
25
20
35
25
20

## Slide 6
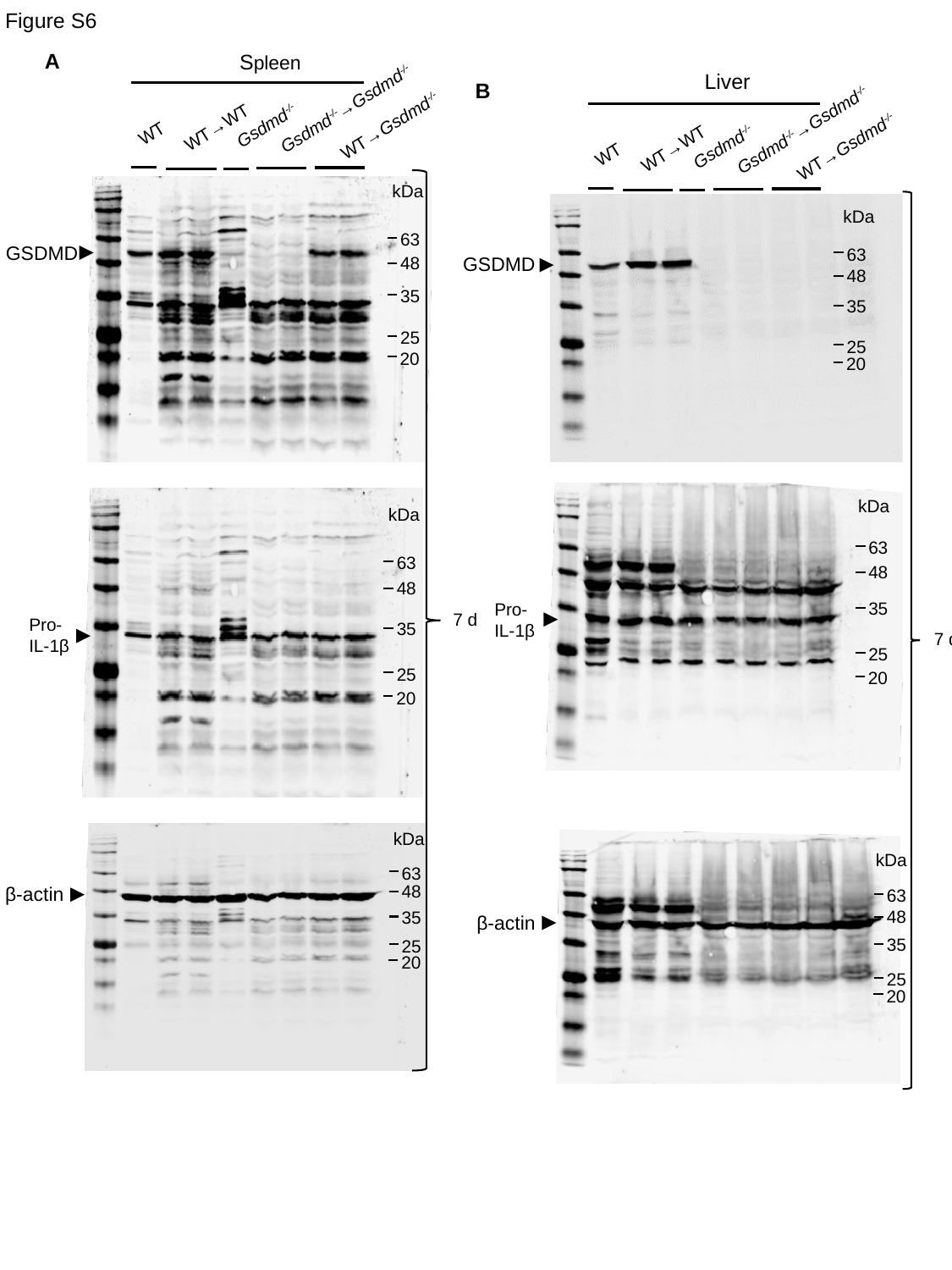

Figure S6
A
Spleen
Liver
B
Gsdmd-/-→Gsdmd-/-
WT→Gsdmd-/-
WT→WT
Gsdmd-/-
Gsdmd-/-→Gsdmd-/-
WT
WT→Gsdmd-/-
WT→WT
Gsdmd-/-
WT
 kDa
 kDa
63
GSDMD
63
GSDMD
48
48
35
35
25
25
20
20
 kDa
 kDa
63
63
48
48
35
Pro-IL-1β
7 d
Pro-IL-1β
35
7 d
25
25
20
20
 kDa
 kDa
63
48
β-actin
63
48
35
β-actin
35
25
20
25
20

## Slide 7
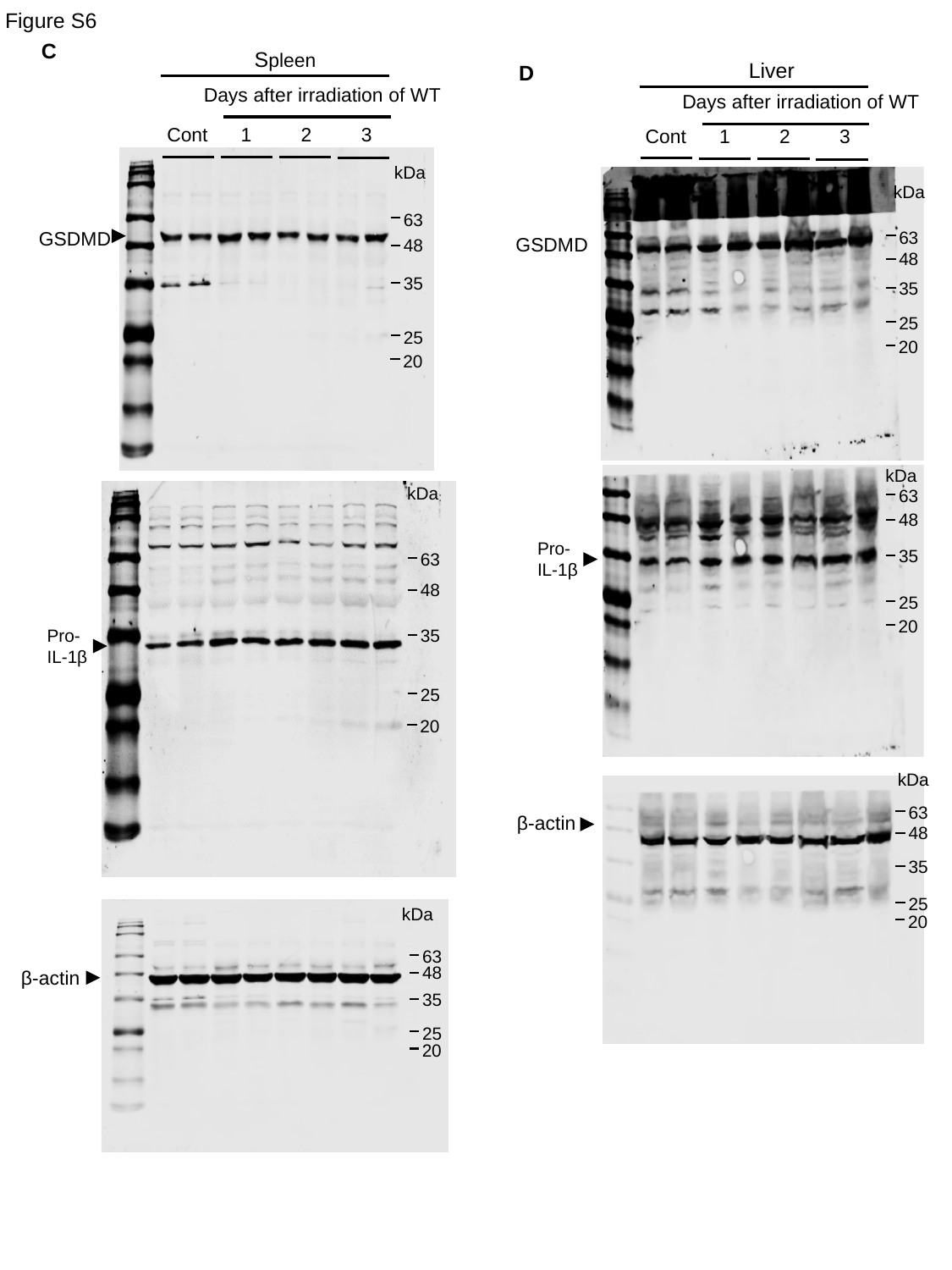

Figure S6
C
Spleen
Liver
D
Days after irradiation of WT
Days after irradiation of WT
Cont 1 2 3
Cont 1 2 3
 kDa
 kDa
63
63
GSDMD
GSDMD
48
48
35
35
25
25
20
20
 kDa
 kDa
63
48
Pro-IL-1β
35
63
48
25
20
Pro-IL-1β
35
25
20
 kDa
63
β-actin
48
35
25
 kDa
20
63
48
β-actin
35
25
20
